# Forward and reverse mutations in stages of cancer development

**DOI:** 10.1186/s40246-018-0170-6

**Published:** 2018-08-22

**Authors:** Taobo Hu, Yogesh Kumar, Iram Shazia, Shen-Jia Duan, Yi Li, Lei Chen, Jin-Fei Chen, Rong Yin, Ava Kwong, Gilberto Ka-Kit Leung, Wai-Kin Mat, Zhenggang Wu, Xi Long, Cheuk-Hin Chan, Si Chen, Peggy Lee, Siu-Kin Ng, Timothy Y. C. Ho, Jianfeng Yang, Xiaofan Ding, Shui-Ying Tsang, Xuqing Zhou, Dan-Hua Zhang, the International Cancer Genome Consortium, En-Xiang Zhou, Lin Xu, Wai-Sang Poon, Hong-Yang Wang, Hong Xue

**Affiliations:** 1 Division of Life Science, Applied Genomics Centre and Centre for Statistical Science, Hong Kong University of Science and Technology, Clear Water Bay, Kowloon, Hong Kong, China; 2 Department of General Surgery, The Second Xiangya Hospital, Central South University, Changsha, Hunan China; 3 Department of Surgery, The Chinese University of Hong Kong, Shatin, Hong Kong, China; 4 Eastern Hepatobiliary Surgery Institute, Second Military Medical University, Shanghai, China; 5 Department of Oncology, Nanjing First Hospital, Nanjing Medical University, Nanjing, China; 6 Jiangsu Key Laboratory of Cancer Molecular Biology and Translational Medicine, Jiangsu Cancer Hospital, Nanjing, China; 7 Division of Neurosurgery, Department of Surgery, Li Ka Shing Faculty of Medicine, Queen Mary Hospital, The University of Hong Kong, 102 Pokfulam Road, Pokfulam, Hong Kong, China; 8 School of Basic Medicine and Clinical Pharmacy, China Pharmaceutical University, Nanjing, China

**Keywords:** Single-nucleotide variation, Copy number variation, Interstitial loss of heterozygosity, Precancer mutations, Clonal evolution

## Abstract

**Background:**

Massive occurrences of interstitial loss of heterozygosity (LOH) likely resulting from gene conversions were found by us in different cancers as a type of single-nucleotide variations (SNVs), comparable in abundance to the commonly investigated gain of heterozygosity (GOH) type of SNVs, raising the question of the relationships between these two opposing types of cancer mutations.

**Methods:**

In the present study, SNVs in 12 tetra sample and 17 trio sample sets from four cancer types along with copy number variations (CNVs) were analyzed by AluScan sequencing, comparing tumor with white blood cells as well as tissues vicinal to the tumor. Four published “nontumor”-tumor metastasis trios and 246 pan-cancer pairs analyzed by whole-genome sequencing (WGS) and 67 trios by whole-exome sequencing (WES) were also examined.

**Results:**

Widespread GOHs enriched with CG-to-TG changes and associated with nearby CNVs and LOHs enriched with TG-to-CG changes were observed. Occurrences of GOH were 1.9-fold higher than LOH in “nontumor” tissues more than 2 cm away from the tumors, and a majority of these GOHs and LOHs were reversed in “paratumor” tissues within 2 cm of the tumors, forming forward-reverse mutation cycles where the revertant LOHs displayed strong lineage effects that pointed to a sequential instead of parallel development from “nontumor” to “paratumor” and onto tumor cells, which was also supported by the relative frequencies of 26 distinct classes of CNVs between these three types of cell populations.

**Conclusions:**

These findings suggest that developing cancer cells undergo sequential changes that enable the “nontumor” cells to acquire a wide range of forward mutations including ones that are essential for oncogenicity, followed by revertant mutations in the “paratumor” cells to avoid growth retardation by excessive mutation load. Such utilization of forward-reverse mutation cycles as an adaptive mechanism was also observed in cultured HeLa cells upon successive replatings. An understanding of forward-reverse mutation cycles in cancer development could provide a genomic basis for improved early diagnosis, staging, and treatment of cancers.

**Electronic supplementary material:**

The online version of this article (10.1186/s40246-018-0170-6) contains supplementary material, which is available to authorized users.

## Background

The progressive development of cancer has been investigated extensively at the cytochemical and genetic levels, leading to the recognition of early premalignant stages characterized by precancerous changes in DNA sequence, gene expression, protein structure, and microscopic rearrangement [1–14]. Genomic analysis also has played an increasingly important role in this regard [15, 16]. In a recent study, we have reported the finding of not only the commonly encountered single-nucleotide variations (SNVs) in the form of gain of heterozygosities (GOHs), but also massive SNVs in the form of interstitial loss of heterozygosities (LOHs) in various types of cancers [17]. This raises the question of the interrelations between the LOH and GOH mutations along with the copy number variations (CNVs) as the most abundant mutational elements of cancer cells. Because cancer cells at different stages of development are known to harbor different mutations, the aim of the present study was to track both GOHs mutating germline homozygous sequence positions to heterozygous ones and LOHs mutating germline heterozygous sequence positions to homozygous ones, through precancer stages to their final allelic forms in the cancer genome.

While forward mutations converting wildtype sequences into mutant forms and reverse mutations restoring the wildtype sequences from the mutant forms have been compared in microbial studies regarding their differential sensitivities to various mutagens [18–20], such studies have not been performed with cancer cells. In this study, a mutation from the original homozygous or heterozygous genotype at a base position in the individual’s germline genome to a different genotype constitutes a forward mutation, and its mutation back to the germline original genotype constitutes a reverse mutation. With the large numbers of GOH and LOH occurrences in cancer cells, it becomes useful to examine whether forward GOHs occurring at one stage of cancer development could be reversed by LOHs during a subsequent stage, and vice versa, during cancer development and enquire into the significance of such reversals. Since premalignant cells have been detected in various instances in the vicinity of tumor cells [2–7, 11, 13], one possible experimental approach would be to analyze and compare solid tumors with their vicinal tissues that might be enriched in precancerous cells in terms of the mutations they harbor. A residue-by-residue analysis of the GOHs and LOHs observed in the tumor and its vicinal tissues relative to the white blood cell genome sequence as a control would reveal GOH-to-LOH and LOH-to-GOH reversals between the germline genotype, any precancerous genotypes, and the cancerous genotype. The same applies to the forward and reverse changes in CNVs.

Accordingly, in the present study, “nontumor” tissue isolated at > 2 cm from the tumor, “paratumor” tissue isolated at ≤ 2 cm from the tumor, and tumor from different types of cancers were compared with same-patient white blood cell controls based on massively parallel sequencing. Somatic mutations in both directions, i.e., GOH and LOH types of SNVs and CNV gains and losses, were examined residue-by-residue and window-by-window in order to detect the presence of mutation reversals during the development of cancer cells and to assess their biological significance. The results obtained from both clinical cancer samples and cultured HeLa cells indicated that forward-reverse (FR) mutations together with directional selection constitute important determinants of the mutation profiles of stage-specific cell populations in cancer development.

## Methods

### Tumor purity and histology

Tumor purity in all B-N-P-T tetra and B-N-T trio samples was estimated using VarScan software [21] and “absCNseq” R package [22]. The “my.res.list” function of absCNseq was applied with the following parameters: alpha.min = 0.2, alpha.max = 1, tau.min = 1.5, tau.max = 5, min.sol.freq = 0, min.seg.len = 0, qmax = 7, and lambda = 0.5.

**Fig. 1:**
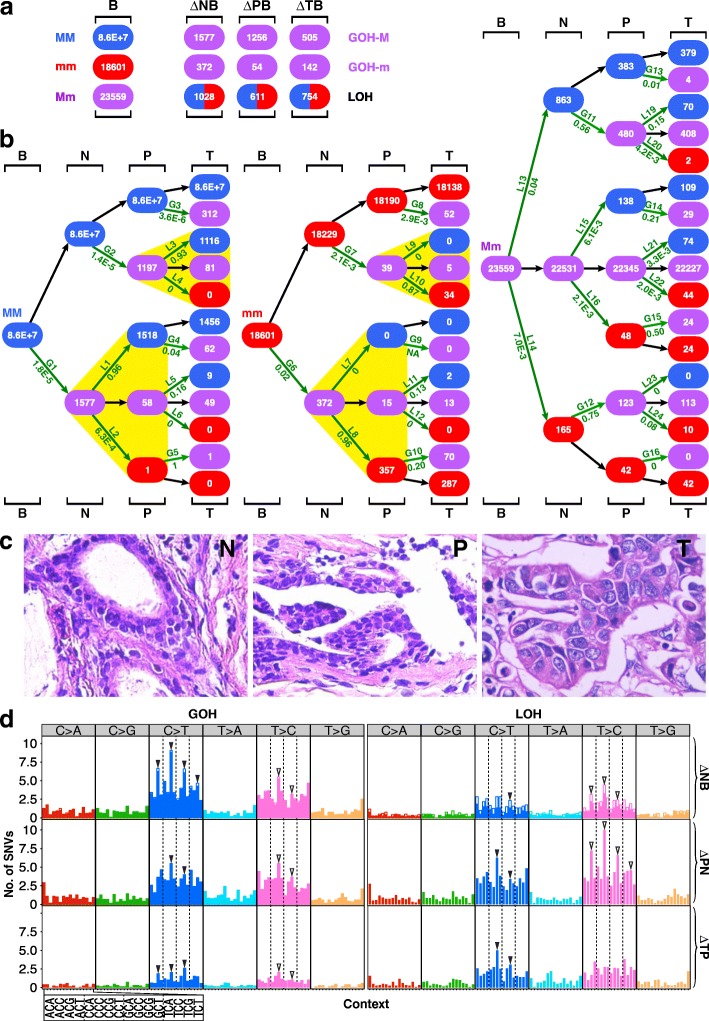
SNV mutations in B-N-P-T tetra samples. **a** Genotypic changes at N-, P-, or T-stage of the samples. The numbers of genotypic changes in N-, P-, or T-stage sequences relative to B-stage sequences are represented by ΔNB, ΔPB, and ΔTB, respectively. LOH represents the sum of LOH-M and LOH-m changes. The 12 B-N-P-T cases consisted of 4 breast carcinomas (BRCA), 5 stomach adenocarcinomas (STAD), and 3 hepatocellular carcinomas (LIHC) analyzed using AluScan sequencing (Additional file 1: Table S1). **b** Patch diagrams tracing SNVs between the B-, N-, P-, and T-samples originating from MM, mm, or Mm genotypes in B-samples. Mutation rate is indicated below each LOH step (L1, L2, etc.) or GOH step (G1, G2, etc.). **c** Micrographs of N-stage tissue (left), P-stage tissue (middle), and T-stage tissue (right) in one of the representative BRCA B-N-P-T tetra samples. Magnification in each instance was × 400. **d** Mutational profiles for the ∆NB, ∆PN, and ∆TP SNV changes as numerically indicated in the patch diagrams in part **b**. The profiles are separated into the C>A, C>G, C>T, T>A, T>C, and T>G types, where C>A includes both the C-to-A and the complementary G-to-T mutations, etc. Within each type, the 16 possible kinds of sequence contexts are indicated on an expanded scale on the *x*-axis, and the total number of SNVs observed for each kind of trinucleotide sequence contexts is represented by a vertical bar. In each vertical bar in the ∆NB tier, the solid segment represents the SNVs that were reversed in the next ∆PN tier, e.g., C>T GOHs being reversed by T>C LOHs, whereas the open segment represents the unreversed SNVs. Subgroups of contexts are compartmentalized by vertical dashed lines. M, major allele; m, minor allele; GOH-M, MM-to-Mm mutation; GOH-m, mm-to-Mm mutation; LOH-M, Mm-to-MM mutation; LOH-m, Mm-to-mm mutation

For histological and immunohistochemical staining (Fig. 1c and Additional file 1: Table S1), the samples were taken from the tumor, the adjacent paratumor region (≤ 2 cm from a tumor), and the nontumor region (> 2 cm from a tumor) of a breast invasive carcinoma (BRCA) patient. The samples were fixed in 4% paraformaldehyde, dehydrated, embedded in paraffin, sectioned, and subjected to standard hematoxylin and eosin (HE) staining. Immunohistochemical staining for estrogen receptor (ER), progesterone receptor (PR), and human epidermal growth factor receptor-2 (HER2) were conducted following the conventional procedures as described [23].

### Genomic DNA from clinical samples for AluScan sequencing

DNA extraction and AluScan sequencing library preparation were performed as described previously [17, 24]. White blood cells were treated as representative of germline controls in keeping with the recommendation by The Cancer Genome Atlas (TCGA) project [25]. The N- and P-stage tissues included and subjected to AluScan sequencing in this study were obtained as follows: N-stage tissue was collected at > 2 cm from the edge of the tumor in the vicinity of the tumor, and P-stage tissue was collected at ≤ 2 cm from the edge of the tumor. In line with the published research practices [5, 6, 26], 2 cm was chosen as the cutoff between N- and P-stage tissues. The AluScan cancer cases, designated as B-N-P-T, B-N-T, or N-T-M sample sets, were listed in Additional file 1: Table S1 with demographical and clinical information.

### Genomic DNA from cultured HeLa cells for AluScan sequencing

HeLa cell line was obtained from American Type Culture Collection (ATCC, USA). Cells were cultivated in DMEM media supplemented with 10% heat-inactivated fetal bovine serum (Sigma-Aldrich, USA), and medium pH was adjusted to 7.4 by 3-(*N*-morpholino) propane sulfonic acid (MOPS) from Sigma-Aldrich, USA. Cultures were incubated at 37 °C in a humidified environment containing 5% CO_2_. To start the HeLa cell culture, frozen cells were thawed and plated at a density of approximately 4 × 10^5^ cells per Petri dish and allowed to reach confluence whereupon they were harvested by treatment with trypsin-EDTA (Gibco, USA), and 10^5^ cells were replated every 2 to 3 days on fresh Petri dishes at intervals of 2 to 3 days. Genomic DNA was isolated from the cells harvested on days 1, 3, 5, 8, 10, 12, 14, 16, 21, 25, 27, and 29 by extraction with TES (100 mM Tris-HCl pH 7.4, 200 mM NaCl, 5 mM EDTA, 0.2% SDS), and centrifuged at 12,000 rpm for 10 min at room temperature. The supernatant was transferred to a new vial and precipitated with an equal volume of ethanol. These successive HeLa cell DNA samplings were subjected to AluScan sequencing.

### AluScan sequencing

AluScans of genomic regions flanked by Alu repetitive sequences were obtained by means of inter-Alu PCR as described [17, 24], employing both head-type and tail-type Alu consensus-based primers to ensure capture of a vast number of inter-Alu amplicons. In brief, a 25-μl PCR reaction mixture contained 2 μl Bioline 10× NH4 buffer (160 mM ammonium sulfate, 670 mM Tris-HCl, pH 8.8, 0.1% stabilizer; www.bioline.com), 3 mM MgCl_2_, 0.15 mM dNTP mix, 1 unit Taq polymerase, 0.1 μg DNA sample, and 0.075 μM each of the four following Alu consensus sequence-based PCR primers:

- AluY278T18 (5′-GAGCGAGACTCCGTCTCA-3′);
- AluY66H21 (5′-TGGTCTCGATCTCCTGACCTC-3′);
- R12A/267 (5′-AGCGAGACTCCG-3′);
- L12A/8 (5′-TGAGCCACCGCG-3′).

PCR was carried out at 95 °C, 5 min for DNA denaturation, followed by 30 cycles each of 30 s at 95 °C, 30 s at 50 °C, and 5 min at 72 °C, plus finally another 7 min at 72 °C. Amplicons were purified with ethanol precipitation, sequenced on the Illumina HiSeq platform at Beijing Genomics Institute (Shenzhen, China) and mapped to the hg19 reference human genome for downstream bioinformatic analysis.

### WGS and WES raw data

Whole-genome sequencing (WGS) data generated from tumor-blood paired samples with the Illumina system by the International Cancer Genome Consortium (ICGC) and TCGA were downloaded in bam format with permission (https://www.synapse.org/#!Synapse:syn2887117). These included the Pilot-63 set and 86 hepatocellular carcinoma (LIHC), 75 non-small-cell lung cancer (NSCLC), and 22 intrahepatic cholangiocarcinoma (ICC) cases with information accessible through the ICGC Data Portal (https://dcc.icgc.org). In addition, raw WGS data generated by Ouyang et al. [27] from four hepatitis B-positive LIHC patients having pulmonary metastasis were obtained along with data from same-patient liver tissue controls and included in the N-T-M trio sample analysis as the WGS-Liver-M subset. Moreover, the raw data of whole-exome sequencing (WES) from 67 brain metastatic cancer patients were obtained from Brastianos et al. [28]. Tissues sampled at > 2 cm from the edge of the tumors were used as normal control tissues in Ouyang et al. [27] and Brastianos et al. [28], and treated as nontumor or N-stage, samples in this study.

### SNV calling

For the paired-end sequencing reads generated on the Illumina platform by the AluScan, WGS, or WES methods, bioinformatics analysis including alignment, sorting, recalibration, realignment, and removal of duplicates using BWA (Burrows-Wheeler Aligner, version 0.6.1) [29], SAMtools (Sequence Alignment/Map, version 0.1.18) [30] and GATK (Genome Analysis Tool-Kit, version 3.5) were performed for the identification of single nucleotide variations (SNVs) according to the standard framework [31] as described previously [17, 24]. The “UnifiedGenotyper” module of GATK was employed for genotyping of SNVs. Only genomic sequence regions with enough coverage, i.e., read depth > 8, were included in the analysis, and the following parameters were applied to filtrate for SNVs of different genotypes: major allele frequency ≥ 95% for the “MM” loci; major allele frequency ≥ 30% and ≤ 70% and QD ≥ 4 for “Mm” or “mn” loci; and minor allele frequency ≥ 95% and QD ≥ 20 for “mm” loci. Strand bias estimated using Fisher’s exact test (FS) was employed to ensure FS value ≤ 20 for both heterozygous “Mm” or “mn” loci and homozygous “MM” or “mm” loci.

For cancer cases with more than two samples from each patient, i.e., the B-N-P-T tetra sample set of 12 cases, the B-N-T trio sample set of 17 cases, and the N-T-M trio sample set of 23 cases, the abovementioned calling of SNVs was first performed for each sample of each case in the multiple sample sets. For each of the multiple-sample cases, only nucleotide positions conformed to all the above SNV calling criteria in every sample of the same patient were included in further analysis. Sites not covered in the further analysis were arising from either lack of sequencing reads or failure to meet the filtering criteria in any one of the samples of the same patient.

### Mutational profiles of SNVs

Mutational profiles of SNVs were analyzed following the procedure developed by Alexandrov et al. [32]. For each SNV site, the SomaticSignatures package [33] under R environment was employed to determine its preceding and following bases. The results were unnormalized for the observed trinucleotide frequencies in the human genome. The resulting mutation frequency profiles were illustrated in three different graphical presentations, i.e., the alteration-group plot, context-group plot, and mutation-rate diagram. Custom R scripts for drawing the three different presentations are available at GitHub website (https://github.com/hutaobo/ProfilePlots).

### CNV calling and identification of recurrent CNVs

From AluScan data, the AluScanCNV software [34] was employed to call paired CNVs between B- and N-stage (∆NB), between N- and P-stage (∆PN), between P- and T-stage (∆TP), between B- and P-stage (∆PB), and between B- and T-stage (∆TB) samples of the same patient in the B-N-P-T tetra sample sets of 12 cases, using fixed window sizes ranging from 50 to 500 kb. The ∆NB, ∆PB, and ∆TB were arranged sequentially to yield the 26 possible serial orders shown in Fig. 8a, b. To identify the recurrent CNVs, all CNVs found in any sequence window of the 12 tetra sample cases at any two of the stages, including ∆NB, ∆PB, ∆TB, ∆PN, and ∆TP, were aggregated. Only the sequence windows where CNV was detected in 6 or more of the 12 patients were considered to harbor a recurrent CNV. CNVs located in the recently identified distal zones [35] were removed from further analysis to reduce background noise introduced by less informative windows in the human genome.

### Co-localization of CNVT with CpGe and MeMRE

CpGe and MeMRE entries were downloaded from UCSC Genome Browser as described [35], and somatic CNV (CNVT) entries classified as “copy number variants” were downloaded from the COSMIC database (http://grch37-cancer.sanger.ac.uk/cosmic/download). The human genome was divided into tandem 2000 bp windows, and the average densities of CNVT breakpoints and base pairs in CpGe or MeMRE in each window were calculated. Thereupon, the windows with zero CpGe or MeMRE density were removed to avoid error caused by missing data, and the remaining windows were separated into ten groups based on the percentile of CpGe or MeMRE density. Finally, the average CNVT breakpoint densities in the groups were plotted against the percentile CpGe or MeMRE density.

### Mutation enrichment in genes and pathways

The results of variant analysis of AluScan data of the 12 tetra sample cases were uploaded to BioMart of the Ensembl database to generate a list of their gene contents under R environment using the “biomaRt” R package [36]. For the “getBM” function, “chromosome_name,” “start_position,” “end_position,” “external_gene_name,” “ensembl_gene_id.” and “description” were selected as attributes, with “chromosomal_region” filter type, “sublist” filter value, “ENSEMBL_MART_ENSEMBL” biomart type, and “grch37.ensembl.org” host. The resultant gene list was uploaded to DAVID Bioinformatics Resources 6.7 [37] using “Functional Annotation Tool” to obtain three lists of mutation enriched functional groups and pathways as annotated in the three databases GOTERM, InterPro, and KEGG, respectively, with mutated genes specified for each group and pathways. Only those functionally annotated groups and pathways yielding Bonferroni-corrected *p* value, Benjamini-corrected *p* value, and FDR *q* value all less than 0.05 were considered statistically significant.

### Statistical analysis and data visualization

Statistical analyses were performed using R software (http://www.r-project.org). The significance probability (*p*) values were calculated by the two-tailed *t* test or chi-square test functions in R, and the Pearson correlation coefficients (*r*) were calculated by the cor function in R. Figures were drawn using the ggplot2, lattice, or ellipse package under R environment, except for Fig. 8b which was drawn using the Circos program [38].

## Results

### Genotypic changes in nontumor and paratumor tissues

White blood cells (B), tumor tissue (T), paratumor tissue (P) immediately adjacent to the tumor, and more remote nontumor tissue (N) were collected in 12 same-patient tetra sample cases consisted of four breast carcinomas (BRCA), five stomach adenocarcinomas (STAD), and three hepatocellular carcinomas (LIHC) (Additional files 1 and 2: Tables S1 and S2) and subjected to DNA analysis using the AluScan platform based on inter-Alu polymerase chain reaction (PCR) followed by massively parallel sequencing as described in the “Methods” section. The genotype of a base residue was referred to as a major allele (M) when it matches the sequence of human reference genome hg19 or as a minor allele (m) when there was no match, thereby enabling the identification of changes in the form of MM-to-Mm GOH (“GOH-M”), mm-to-Mm GOH (“GOH-m”), Mm-to-MM LOH (“LOH-M”), or Mm-to-mm LOH (“LOH-m”) [17]. Figure 1a, b show the total number of residue-by-residue changes in the N-, P-, or T-sample genomes relative to B-sample, viz. ∆NB, ∆PB, or ∆TB respectively in terms of GOH-M, GOH-m, and LOH (sum of instances of LOH-M and LOH-m). Since the numbers of GOH-M, GOH-m, and LOH mutations were higher in ΔNB than in ΔTB, and comparable in ΔPB and ΔTB, both the N-sample and P-sample cells had to be regarded as premalignant or early malignant cells despite their normal morphology and expression of immunohistochemistry (IHC) markers, in contrast with T-sample cells showing enlarged nuclei (Fig. 1c) and reduced expression of IHC markers (Additional file 1: Table S1). Since the residues of minor bases different from “m” were rare in the samples analyzed, mutations involving them are listed in Additional files 3 and 4: Tables S3 and S4 but not shown in Fig. 1a, b. Notably, 96% of the B-to-N-stage forward GOH mutations were reversed in P-stage via steps L1 and L8, and 56% and 75% of the B-to-N-stage forward LOHs were reversed via steps G11 and G12, respectively. On the other hand, only 16% and 0% of the B-to-N-stage forward GOH mutations were reversed in T-stage via steps L5 and L12, and only 1% and 0% of the B-to-N-stage LOH mutations were reversed in T-stage via steps G13 and G16.

Moreover, the LOH mutations partitioned Mm genotypes between MM and mm products on a non-random basis. Thus, the ratio of MM/mm products from the L1 and L2 steps was 1518/1, whereas the ratio of MM/mm products from the L7 and L8 steps was 0/357. Likewise, the ratio of MM/mm products from the L3 and L4 steps was 1116/0, and the ratio of MM/mm products from the L9 and L10 steps was 0/34. Therefore, the partition of LOH products in each of these instances was biased by strong lineage effect in favor of restoring the original germline genotype that gave rise to the Mm residue in the first place (as highlighted by yellow triangles in Fig. 1b).

**Fig. 2:**
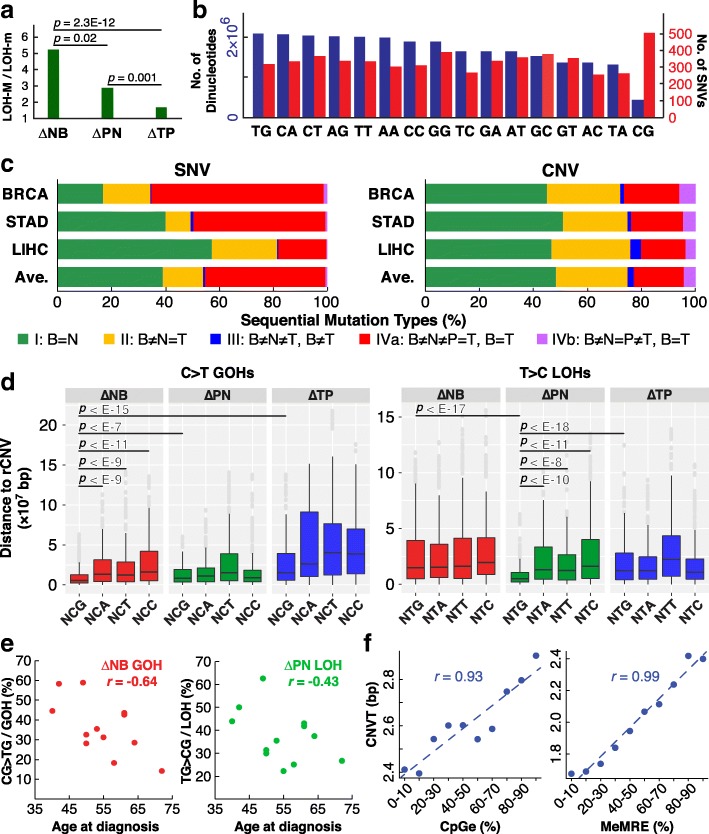
Properties of mutations in B-N-P-T tetra samples. **a** M over m preference in LOHs. LOH-M/LOH-m ratios for LOHs arising at germline Mm positions are shown for N-, P-, and T-stages (data from Fig. 1b, right panel). **b** Dinucleotides in genomic sequences captured by AluScan (blue bars) and SNVs found at the first base of dinucleotides (red bars). **c** Percentile of different types of sequential SNV and CNV changes in 12 tetra sample cases of BRCA, STAD, and LIHC. Type I (green) B=N, viz. no SNV (or CNV) found in ∆NB. Type II (yellow) B ≠ N = T, viz. same SNV (or CNV) found in ∆NB and ∆TB. Type III (blue) B ≠ N ≠ T and B ≠ T, viz. altered in ∆NB and ∆TN and also in ∆TB. Type IVa (red) B ≠ N ≠ P = T and B = T, viz. altered in ∆NB and ∆PN but not in ∆TP or ∆TB. Type IVb (purple) B ≠ N=P ≠ T and B = T, viz. altered in ∆NB and ∆TP but not in ∆PN or ∆TB. **d** Average distances between GOHs (left) or LOHs (right) and their nearest recurrent CNVs (rCNVs). The left panel shows that on average, CG>TG GOHs in ∆NB were closer to their nearest rCNVs than it was the case with 11 other kinds of C>T GOHs; the right panel shows that on average, TG>CG LOHs in ∆PN were closer to their nearest rCNVs than it was the case with 11 other kinds of LOHs, with the horizontal bars indicating how much closer in terms of *p* values. **e** Correlation between patient’s age at diagnosis and percentage of CG>TG GOHs among all GOHs for ∆NB (left) or percentage of TG>CG LOHs among all LOHs for ∆PN (right). **f** Correlation of numbers of somatic CNV breakpoints found in tumors (CNVT) from the COSMIC database with either evolutionarily conserved CpG-rich regions (CpGe) (left panel) or unmethylated CpG-rich regions (MeMRE) (right panel) from UCSC Table Browser database [35]. *p*, significance probability; *r*, Pearson correlation coefficient (see Fig. 1 for abbreviations)

Notably, in Fig. 1b right panel, the partition of the germline Mm genotypes via LOH steps L13 and L14 yielded a greater MM/mm product ratio than the partition via LOH steps L15 and L16, and greater still than the partition via LOH steps L21 and L22, although in each instance, MM products exceeded mm products (Fig. 2a). Since all these three successive partitions emanated from the germline Mm genotypes, their diminishing MM/mm ratios could not be the consequence of lineage effects. Instead, because MM genotypes in the genome have been optimized in general for growth in the course of human evolution, they tended to be favored over mm genotypes. The finding of [L13/L14 = 5.7] > [L15/L16 = 2.9] > [L21/L22 = 1.7] could be the result of the N-stage cells having gone through a more prolonged period of positive selection for MM genotypes than the P-stage cells, and the P-stage cells in turn have gone through a more prolonged period of positive selection than T-stage cells.

When the trinucleotide-based mutational profile method [32] was employed to classify the GOHs and LOHs observed in the B-N-T-P tetra samples into the C>A, C>G, C>T, T>A, T>C, and T>G groups, the results showed that C>T and T>C mutations were particularly prominent among both GOHs and LOHs, in keeping with the expectation that transitions would exceed transversions in SNVs (Fig. 1d). The C>T GOHs among the ∆NB changes displayed peak frequencies at the four NCG triplets, conforming to the “signature 1A” (marked by four solid arrowheads) common to cancers, and likely ascribable to the contribution of spontaneous deamination of 5-methylcytosine at methylated CpG to form thymidine [32, 39]. These deaminations would also explain the ~ 50% greater occurrence of CG>TG GOHs than TG>CG GOHs in the ∆NB changes. In support of this, Fig. 2b shows that although there were less CG dimers than other dimers among AluScan captured as well as whole-genome sequences (Additional file 5: Figure S1), more CG dimers underwent SNV mutations than any other dimers. In Fig. 1d, all SNV frequency columns in the ∆NB tier were represented by a solid segment and an open segment; the mutations in the solid segments were reversed in the next ∆PN tier, whereas the open segments were unreversed. Both the C>T and T>C GOHs show large solid segments indicating their extensive reversals in the ∆PN changes; since the T>C LOHs in the ∆PN tier were mostly reversals of the C>T GOHs in the ∆NB tier, these T>C LOHs were likewise more abundant than ∆PN C>T LOHs and showed four NTG peaks (marked by open arrows), which may be referred to as a “signature 1A”-like LOH feature.

Figure 2c summarizes the forward and reverse mutation occurrences in the B-N-P-T samples; more SNVs and CNVs occurred in N-stage (viz. sum of types II, III, IVa, and IVb patterns) than in P- and T-stages combined (viz*.* type I). Reversals of N-stage SNVs and CNVs (viz. sum of types IVa and IVb patterns) were common, amounting to ~ 70% of N-stage SNVs or ~ 40% of N-stage CNVs, and far more of such reversals took place in P-stage (type IVa) than in T-stage (type IVb), see Additional files 3 and 6: Tables S3 and S5 for detailed numbers of the SNVs and CNVs at different stages.

**Fig. 3:**
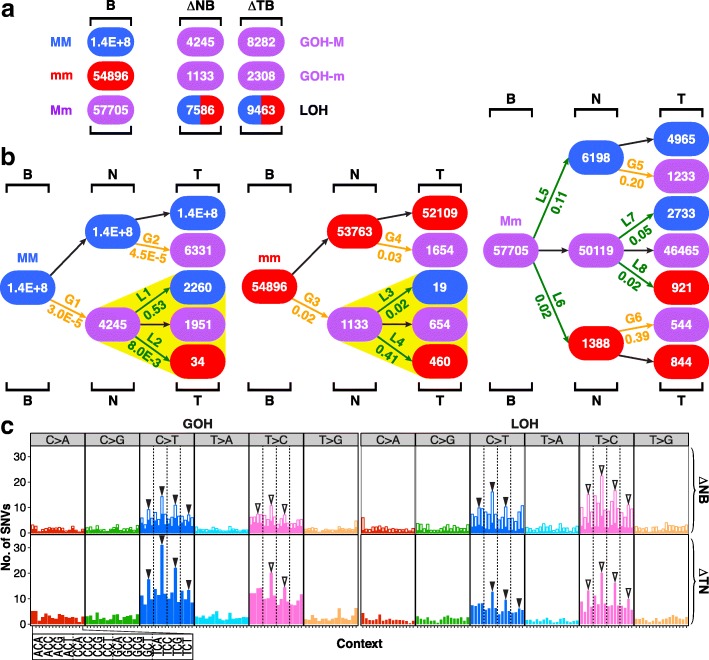
SNV mutations in B-N-T trio samples. **a** Genotypic changes in N- or T-stage of the samples. The numbers of genotypic changes in N- or T-stage sequences relative to B-stage sequences are represented by ΔNB and ΔTB, respectively. The 17 B-N-T trio samples consisted of 1 BRCA, 2 LIHC, and 14 non-small cell lung cancer (NSCLC) cases. **b** Patch diagrams tracing SNVs between the B-, N-, and T-samples. **c** Mutational profiles for the ∆NB and ∆TN SNV changes as numerically indicated in the patch diagrams in part **b**. In each vertical bar in the ∆NB tier, the solid segment represents the SNVs that were reversed in the ∆TN tier, whereas the open segment indicates the unreversed SNVs (see Additional files 7 and 8: Tables S6 and S7 for details of SNVs, and Fig. 1 for abbreviations)

When another 17 B-N-T trio sample sets consisted of 1 BRCA, 2 LIHC, and 14 non-small cell lung cancers (NSCLC) were analyzed with respect to the GOH and LOH changes in the N- and T-stage cells relative to B-stage cells (Fig. 3, Additional files 7 and 8: Tables S6 and S7), the results obtained showed the same regularities as the B-N-P-T tetra samples: the B genomes displayed much higher LOH (L5, L6) rates and GOH-m (G3, G4) rates than GOH-M (G1, G2) rates, strong lineage effects in LOH partitions between MM and mm products (highlighted by yellow triangles), and prominent FR-mutations, viz. L1 reversing G1, L4 reversing G3, G5 reversing L5, and G6 reversing L6.

### Genotypic changes in cultured HeLa cells

**Fig. 4:**
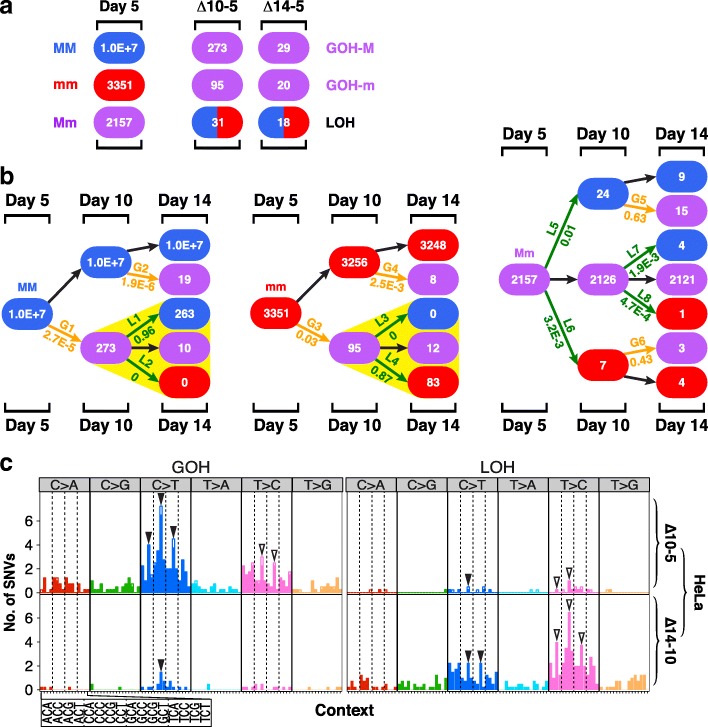
SNV mutations in HeLa cells. **a** Numbers of base positions of different genotypes on day 5, and their changes observed on day 10 relative to day 5 (Δ10–5), and on day 14 relative to day 5 (Δ14–5). **b** Patch diagrams tracing the mutated changes of MM, mm, and Mm residues of day 5 DNA in day 10 DNA and day 14 DNA. LOHs showing large lineage effects are highlighted by yellow triangles. **c** Mutational profiles for the changes indicated in part **b** between day 5 and day 10 DNAs (∆10–5) and between day 10 and day 14 DNAs (∆14–10). For each vertical bar in the ∆10–5 tier, the solid segment represents SNVs that were subsequently reversed in the ∆14–10 tier, and the open segment represents the unreversed SNVs

When frozen HeLa cells were restarted in culture and sequentially sampled for AluScan sequencing, the results obtained also showed a wave of forward mutations followed by reverse mutations. Figure 4a shows the changes in the genotypes of base residues between day 10 and day 5 (viz. ∆10–5) and between day 14 and day 5 (viz. ∆14–5), and these changes are indicated in the patch diagrams in Fig. 4b. Notably, of the 273 MM residues that mutated to Mm via the G1 step, 263 of them were reverted to MM by day 14, and none was mutated to mm. Similarly, of the 95 mm residues that mutated to Mm via the G3 step, 83 of them were reverted to mm, and none was mutated to MM. Thus, the ratio of MM/mm products from the yellow-highlighted L1 and L2 LOH steps was 263/0 and that for the L3 and L4 LOH steps was 0/83, displaying striking lineage effects in both instances comparable to the lineage effects displayed by the N-stage cells in Fig. 1b that were also yellow-highlighted. Since HeLa cells were transformed cells, the forward-reverse mutation cycles formed by the G1-L1 steps, or by the G3-L4 steps, in Fig. 4b could not be related to the oncogenic transformation. Instead, they likely represented a mechanism employed by the cells in the process of adapting to replating and growth.

Figure 4c shows the mutational profiles of GOHs (left panel) and LOHs (right panel) observed in the transitions between day 5 and day 10 (viz. ∆10–5, upper tier) and between day 10 and day 14 (viz. ∆14–10, lower tier), where the solid or open segments in the ∆10–5 tier represent the mutations that were reversed or unreversed respectively in the ∆14–10 tier. As in the case of the profiles for the ∆NB and ∆PN changes in Fig. 1d, both the CG>TG (blue) and TG>CG (pink) GOH peaks in the ∆10–5 tier were extensively reversed in the ∆14–10 tier, giving rise to the prominent TG>CG (pink) and CG>TG (blue) LOH peaks respectively in the ∆14–10 tier. The G1, L1, L2, G3, L3, and L4 rates in Fig. 4b were also similar to their counterpart G1, L1, L2, G6, L7, and L8 rates in Fig. 1b.

### Genotypic changes in primary and metastatic tumors

**Fig. 5:**
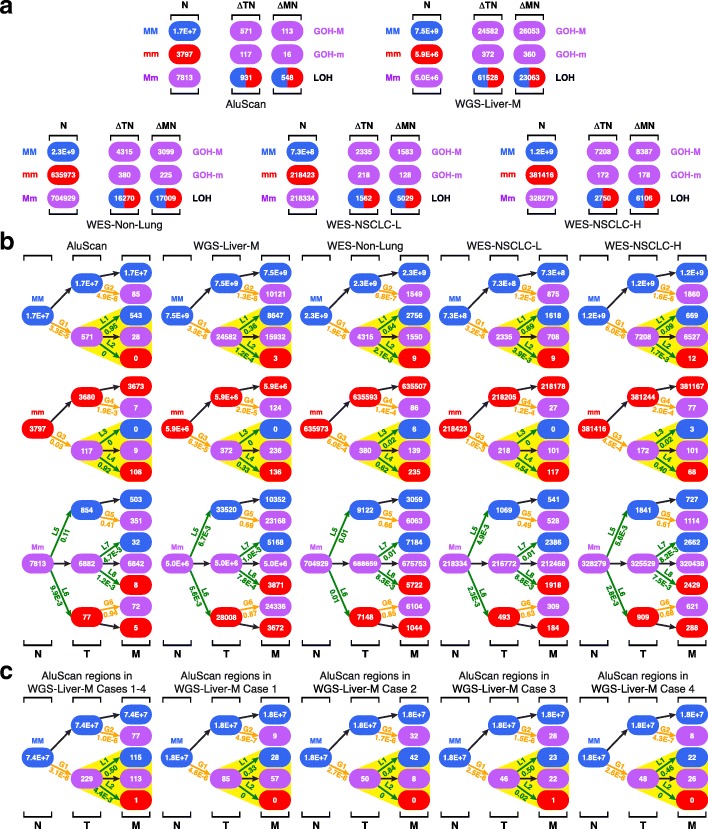
SNV mutations in N-T-M trio samples. **a** Genotypic changes in T- or M-stage sequences. The numbers of genotypic changes in T- or M-stage sequences relative to N-stage sequences in each of the five case groups are represented by ΔTN and ΔMN, respectively. The five case groups include AluScan group of 2 N-T-M trio sets analyzed with AluScan sequencing, WGS-Liver-M group of 4 trio sets of liver-to-lung metastasis analyzed by whole-genome sequencing (WGS), and 67 trio sets involving brain metastases analyzed with whole-exome sequencing (WES) which were separated into 38 WES-Non-Lung cancers, 6 WES-NSCLC-L (L = low in C>A GOHs) cancers, and 23 WES-NSCLC-H (H = high in C>A GOHs) cancers. In the trio sets of WGS-Liver-M, WES-Non-Lung, WES-NSCLC-L, and WES-NSCLC-H, because nontumor tissues were sampled at > 2 cm from tumor’s edge as controls instead of blood cells, the samples were designated as “N-stage” for comparability with Figs. 1a and 3a. **b** Patch diagrams tracing SNVs between the N, T, and M samples (see Additional files 9 and 10: Tables S8 and S9 for details of SNVs). M, metastatic tumor. (liver = hepatocellular carcinoma; NSCLC = non-small-cell lung cancer; non-lung = all 12 types of solid tumors in Reference [27] except lung adenocarcinomas and lung squamous carcinomas, see Fig. 1 for abbreviations). **c** Patch diagrams tracing the SNVs in the fraction of the four WGS sequences in the WGS-Liver-M group that corresponded to all the AluScan-captured regions analyzed in Fig. 1

**Fig. 6:**
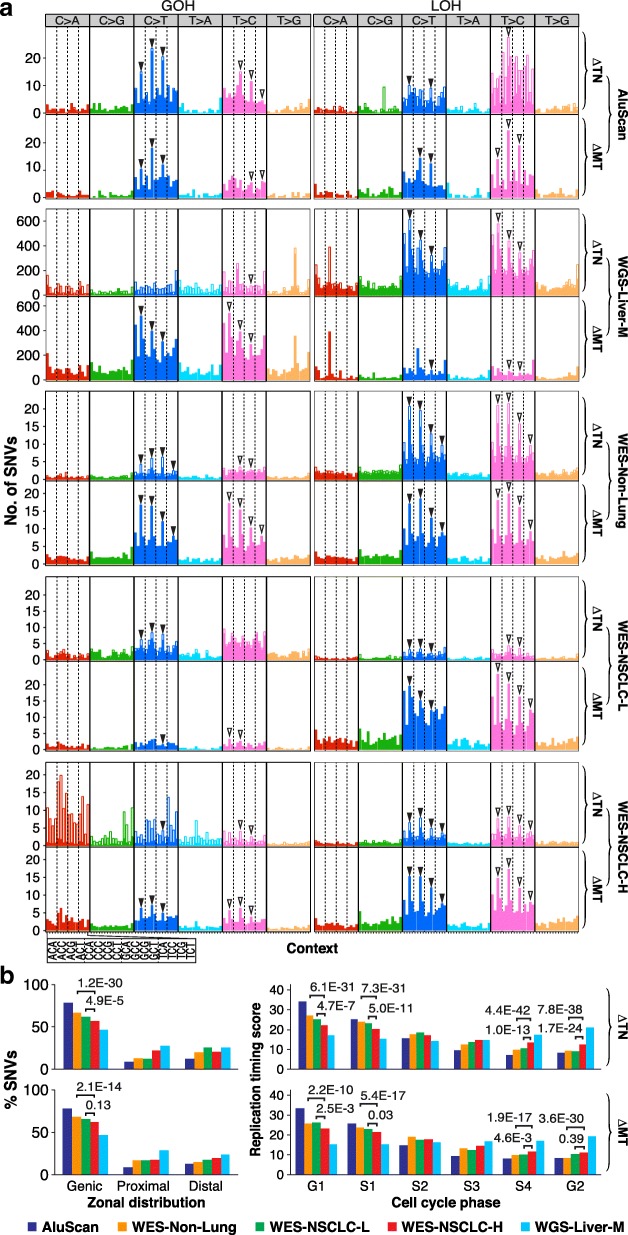
Properties of SNVs in N-T-M trio samples. **a** Mutational profiles for the ∆TN and ∆MT SNV changes as numerically indicated in the patch diagrams in Fig. 5b. In each vertical bar in the ∆TN tier, the solid segment represents the SNVs that were reversed in the ∆MT tier, whereas the open segment indicates the unreversed SNVs. **b** Zonal distribution and replication timing of the SNVs in the ∆TN tier and the ∆MT tier in the five case groups. Left panels: zonal classification of SNV (GOH and LOH) sites determined as described [35]. Right panels: replication timing scores derived from ENCODE at UCSC based on the “Repli-chip” method [52] (see Figs. 1 and 5 for abbreviations)

Figures 5 and 6 compare the mutations observed in five cancer groups based on same-patient N-stage, T-stage, and metastatic stage (M-stage) samples: (i) AluScan group of 2 N-T-M trio sets analyzed with AluScan sequencing, (ii) WGS-Liver-M group of 4 trio sets of liver-to-lung metastasis analyzed by Ouyang et al*.* [27] using WGS, and 67 trio sets involving brain metastases analyzed with WES by Brastianos et al. [28], which were separated into (iii) 38 WES-Non-Lung cancers, (iv) 6 WES-NSCLC-L (L = low in C>A GOHs) cancers, and (v) 23 WES-NSCLC-H (H = high in C>A GOHs) cancers. Although the five N-T-M trio groups compared in Fig. 5a were analyzed using variously the AluScan, WES, and WGS platforms, the ratios of the [∆TN]/N and [∆MN]/N counts both indicated that the rates of LOH far surpassed the rates of GOH-m, which in turn far surpassed the rates of GOH-M (Additioanl files 9 and 10: Tables S8 and S9). All five groups also displayed pronounced lineage effects in Fig. 5b in the partitions of LOH mutations of Mm genotypes between MM and mm products (highlighted by yellow triangles).

In Fig. 6a, the relative prominences of ∆TN GOHs, ∆TN LOHs, ∆MT GOHs, and ∆MT LOHs varied among the five different cancer groups. This could arise in part from biological dissimilarities between the sequences analyzed on the different platforms on account of their varied sequence coverages of the genome. The SNV sites observed in the five groups displayed non-identical distributions among the genic, proximal, and distal sequence zones [35], as well as non-identical replication timings during the cell cycle (Fig. 6b). The proportion of ∆TN GOHs that became reversed in the ∆MT changes, marked by solid segments of the GOH frequency bars in the ∆TN tiers, was highest in the AluScan group, also quite high in the WES-NSCLC-L group, modest in the WES-Non-Lung group, and lowest in the WGS-Liver-M and WES-NSCLC-H groups, even though the WES-NSCLC-L, WES-Non-Lung, and WES-NSCLC-H groups were all analyzed based on the WES platform [28].

The WES-NSCLC-H group was unique in its display of particularly eminent C>A GOHs. Previously, C>A transversions were linked to polycyclic aromatic hydrocarbons [40] and acrolein [41] in tobacco smoke. The 23 WES-NSCLC-H samples were derived entirely from smokers, in accord with smoking being a significant factor for their elevated C>A GOHs. However, the WES-NSCLC-L samples with much more subdued C>A GOHs included two non-smokers and four smokers, suggesting that smoking or high C>A GOHs could play a less important carcinogenic role in a minority of smokers.

When the AluScan-capturable regions were extracted from the four N-T-M trio samples in the WGS-Liver-M group and analyzed with respect to their genotypic changes, the results obtained were similar to those obtained from the entire WGS sequences for the same samples: (a) among the ∆TN changes, the LOH/GOH-M ratios of 2.5 for the WGS-based samples (Fig. 5a), and 2.1 for the AluScan-based samples (Additional file 11: Figure S2a), were both substantially greater than unity; (b) among the ∆MN changes, the LOH/GOH-M ratios of 0.89 for the WGS-based samples, and 1.1 for the AluScan-based samples, were both close to unity; (c) in the patch diagram for the total WGS-based mutations originating from MM residues in the four samples (Fig. 5b), the rates for the L1 and L2 steps were 0.36 and 1.2E−4, respectively. In the total AluScan-based samples (Additional file 11: Figure S2b), the rates for the L1 and L2 steps were 0.50 and 4.4E-3 respectively. Thus, both WGS-based and AluScan-based analyses yielded a high L1/L2 > 100 rate ratio indicative of strong lineage effects in the LOH mutations that reversed the GOH mutation in the G1 step; and (d) the mutational profiles for the AluScan-based ∆TN and ∆MT changes (Additional file 11: Figure S2c) were highly similar to the WGS-based ones (Fig. 6a, WGS-Liver-M) with respect to the major mutation peaks in both the N-to-T and T-to-M transitions. The patch diagrams for the four individual AluScan-based cases (cases 1–4, Fig. 5c) were all similar to that for their sum total (WGS-Liver-M, Fig. 5c) in the much larger numbers of LOHs arising from L1 step compared to L2 step, testifying in each instance to a strong lineage effect.

### Whole-genome sequencing confirmed the abundance of interstitial LOHs

**Fig. 7:**
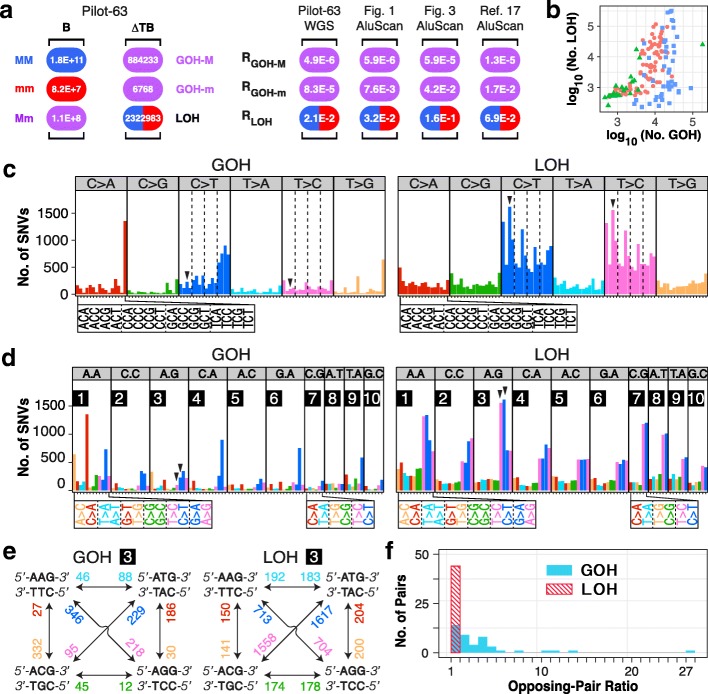
Properties of ICGC whole-genome sequencing of tumor (T)-blood (B) pairs. **a** Left panel: numbers of different genotypic changes between T- and B-stage sequences (∆TB) in ICGC Pilot-63 pan-cancer samples. Right panel: rates of different types of SNV mutations. For Pilot-63 samples, the rates were derived from WGS ∆TB/B values in the left panel; for B-N-P-T AluScan samples, from ∆TB/B values in Fig. 1a; for B-N-T AluScan samples, from ∆TB/B values in Fig. 3a; for Reference [17] samples, from AluScan results on six types of cancers [17]. **b** Scatter plot of the numbers of GOHs and LOHs in 22 intrahepatic cholangiocarcinoma (ICC, green triangles), 86 hepatocellular carcinomas (LIHC, red circles), and 75 non-small cell lung cancer (NSCLC, blue squares) pairs of samples (Additional file 12: Table S10). **c** Alteration-group plots of mutational profiles for GOHs and LOHs between B- and T-stages in Pilot-63 samples. Each plot was grouped using the six alteration types: C>A, C>G, C>T, T>A, T>C, and T>G. **d** Context-group plots of mutational profiles for GOHs and LOHs. Each plot is arranged by the ten context groups: A.A, C.C, A.G, C.A, A.C, G.A, C.G, A.T, T.A, and G.C, designating the different immediate 5′ and 3′ flanking nucleotides. The opposing GOH mutations (left panel) or opposing LOH mutations (right panel) are placed side-by-side (color-coded as in part **c**). **e** Representative mutation-rate diagrams, GOH-3 (left panel) and LOH-3 (right panel), were generated from the ICGC Pilot-63 dataset (see additional nine pairs of diagrams in Additional file 13: Figure S3). In these diagrams based on triplet duplexes, the rates of opposing mutations from part **d** were paired and labeled on bidirectional arrowheads with same color-codes as in part **d**. **f** Distribution of the observed rate ratios between opposing GOH pairs (the blue columns) and LOH pairs (the striped red column) (see Fig. 1 for abbreviations)

A total of 246 tumor-control pairs from the International Cancer Genome Consortium (ICGC) collection of WGS data [42] were analyzed to yield LOH and GOH types of SNVs in each paired samples. These included a panel of 63 pan-cancer cases (pilot-63) (Fig. 7a), 22 intrahepatic cholangiocarcinoma (ICC), 86 LIHC, and 75 NSCLC cases (Fig. 7b, Additioanl file 12: Table S10), showing prominent LOHs in each instance. In the Pilot-63 dataset, the different ΔTB mutation counts in T-stage cells relative to B-stage cells (Fig. 7a left panel) yielded a rate ratio of 4300 between LOH and GOH-M, which was comparable to the 5400, 2700, and 5300 rate ratios observed in Figs. 1 and 3 and earlier in Reference [17], respectively, indicating a vastly greater rate of LOH than GOH in the cancer cells in all four instances. As well, in all four instances, the three mutation rates remain in the same order of R_LOH_ > R_GOH-m_ > R_GOH-M_, with LOH rate being the highest. The massive interstitial LOH rates observed earlier based on AluScan data were thus confirmed by the ICGC Pilot-63 WGS dataset.

### Evidence from mutational profiles for gene conversions in LOH production

For the ∆TB SNVs of Pilot-63 WGS dataset, Fig. 7c shows the alteration-group plots of mutational profiles, which are rearranged in Fig. 7d so that opposing GOH pairs or opposing LOH pairs are placed side-by-side, e.g., by pairing the ATG>ACG GOH (pink bar) with the ACG>ATG GOH (blue bar) in section 3 of the left panel and similarly pairing the ATG>ACG LOH (pink bar) with the ACG>ATG LOH (blue bar) in section 3 of the right panel (marked by arrowheads). Figure 7d shows the strikingly similar heights of the opposing C>T (blue) or T>C (pink) LOH bars in the right panel, but the generally dissimilar heights of the opposing C>T (blue) or T>C (pink) GOH bars in the left panel. The context-group plots in Additional file 13: Figure S3a show comparable rates for different pairs of opposing LOHs in contrast to the generally unequal rates for different pairs of opposing GOHs. The rates for the individual pairs of opposing GOHs are further displayed in the mutation-rate diagrams in Additional file 13: Figure S3a and those for the opposing LOHs in the mutation-rate diagrams in Additional file 13: Figure S3b. One of the GOH diagrams from Additional file 13: Figure S3a and one of the LOH diagrams from Additional file 13: Figure S3b are illustrated in Fig. 7e, showing the frequencies for all opposing GOH pairs or opposing LOH pairs, e.g., the rates of ACG>ATG GOH and ATG>ACG GOH changes (arrowhead-marked in Fig. 7d left panel) were dissimilar (229 in blue versus 95 in pink), but the rates of LOH changes of the same triplet duplexes (arrowhead-marked in Fig. 7d right panel) were similar (1617 in blue versus 1558 in pink). The ratios between the frequencies (or rates) of the different LOH pairs were 192/183, 150/141, 713/704, 1617/1558, 204/200, and 178/174, which varied only between 1.01–1.06. The results from Additional file 13: Figure S3a and b are summarized in Fig. 7f, where over 61% of the opposing pair frequencies were greater than 2 and spread between 1 and 14 for the GOHs (blue bars), but 100% between 1 and 2 for the LOHs (striped red bars), clearly indicative of the different mutational mechanisms employed for the production of the GOHs versus the LOHs. This divergence of the rate ratios between opposing GOHs and opposing LOHs was in accord with our proposal that the LOHs in cancer cells were generated mainly by double-strand break (DSB) repairs through gene conversion, whereas the GOHs were produced by more diverse mechanisms including mutations due to the highly error-prone nature of the DNA polymerase employed for interhomolog recombination [17] and deaminations that accounted for the ~ 50% greater occurrence of CG>TG GOHs than TG>CG GOHs in the ∆NB changes (Fig. 1d). In a DSB at a heterozygous C/T, LOH by gene conversion could yield either a C/C or T/T homozygous position at comparable rates, depending on which homologous chromatid bears the DSB. On the other hand, because GOHs depend on point mutations rather than gene conversions, this comparable-rate constraint would not apply to GOHs.

Moreover, for the B-N-P-T tetra samples, 95.5% of the forward LOHs in the B-to-N transition (steps L13 and L14, Fig. 1b), 98.7% of the reverse LOHs in the N-to-P transition (steps L1 and L8, Fig. 1b), and 95.2% of the reverse LOHs in the P-to-T transition (steps L3 and L10, Fig. 1b) occurred within the copy number neutral regions (Additional file 14: Figure S4), suggesting that both the forward LOHs and the reverse LOHs were mostly brought about by gene conversion.

### Distances between SNVs and recurrent CNVs

That the N-stage SNVs and CNVs in the B-N-P-T tetra samples both underwent active reversions, and more in P-stage than in T-stage (Fig. 2c) suggest some form of possible correlation between these two types of mutations. This was supported by Fig. 2d which shows that the sites of C>T GOHs with NCG context occurring in the ∆NB changes, and T>C LOHs with NTG context occurring in the ∆PN changes, were located particularly close to the recurrent CNVs compared to the mutations with other contexts or in other stages of change, *p* < 10^−7^. Furthermore, these two groups of SNVs declined with the age at diagnosis (Fig. 2e), in resemblance to the decrease of global DNA methylation in old age [43]. The correlation between somatic CNVs with CpGe and MeMRE (Fig. 2f), the increased SNVs at CpG sites (Fig. 2b), and the high tendency of methylated CpG conversion to TpG [44] also pointed to some SNV-CNV relationships in the CNV production process, such as breakpoint misrepair and merit investigation.

### Frequency classes of serial CNV changes

**Fig. 8:**
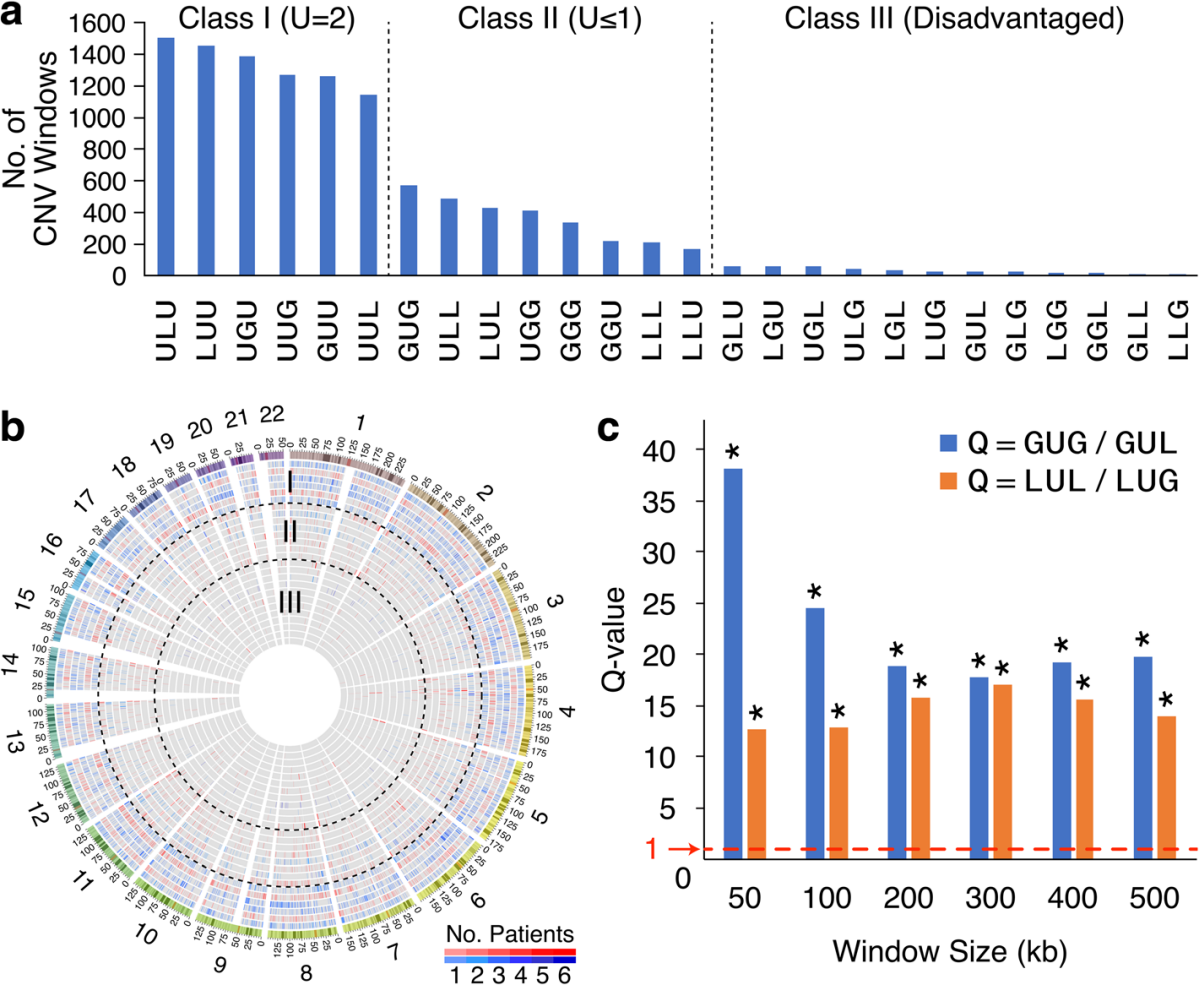
Serial orders of copy number (CN) changes in B-N-P-T tetra samples. **a** Frequencies of three classes of sequential orders of CN changes. Total numbers of CNV windows conformed to each serial order of changes are plotted out for all 26 possible orders. The CN status of 500 kb windows of N-, P-, or T-stage was determined relative to B-stage for 12 B-N-P-T tetra samples as described in the “Methods” section. The total numbers of windows that conformed to the different possible serial orders of CN changes (Additional file 15: Table S11) provided the basis for their partition into three frequency classes (separated by two vertical dashed lines). U stands for CN-unaltered, G for CN-gain, and L for CN-loss. **b** Circos diagram showing the chromosomal distribution of different CNV serial orders. The 26 different orders are arranged from the rim, showing chromosome banding, inward in accordance to their left-to-right arrangement in part **a**, viz. ULU (blue) in the outermost ring followed by LUU (red), UGU (blue), etc., where orders with a CN-status of U at N-stage are colored blue, and orders with a CN-status of G or L at N-stage are colored red. Classes I–III are separated by dashed circles in black. **c** GUG/GUL and LUL/LUG frequency ratios determined using different window sizes. Without lineage effects, these ratios would be expected to yield *Q* = 1 (as represented by the red dashed line). In contrast, all the *Q* values of GUG/GUL (blue) and LUL/LUG (red) observed in sequence windows varying from 50 to 500 kb significantly exceeded unity (*p < 10^−16^) (see Fig. 1 for abbreviations)

In the B-N-P-T tetra sample cases, the status of any CNV in the N-, P-, and T-stages could be CN-unaltered (U), CN-gain (G), or CN-loss (L) relative to its status in B-stage. Arranging in serial order, the CN-status found in the N-P-T stages (Additional file 6 and 15: Tables S5 and S11) yielded 26 different serial orders, and their frequencies fell into three classes (Fig. 8a, b). In the LUG order, for example, each CNV site was CN-loss in N-stage, CN-unaltered in P-stage, and CN-gain in T-stage, and the total number of sequence windows in the B-N-P-T sequences analyzed that exhibited such an LUG order made up the frequency on the *y*-axis of Fig. 8a. The three frequency classes separated by vertical dashed lines in the figure were:

I. Class I (U = 2)—comprising six different orders, where a U status occurred in two of the N-, P-, and T-stages.
II. Class II (U ≤ 1)—comprising eight different orders, where the U status occurred in no more than one of the N-, P-, and T-stages.
III. Class III (disadvantaged)—comprising 12 different orders, where 10 of them (viz. outside of LUG and GUL) included an abrupt double-dose change directly from G to L, or L to G in the order.

The plausible basis for these different classes could be straightforward; the CNV orders in class I entailed minimal copy-number departures from the starting B-stage and were therefore well tolerated; in comparison, the class II of CNV orders incurred greater departures from U and were less well tolerated. Every CNV order in class III involved at least one double-dose change jumping either from G to L or from L to G between two successive stages of cancer development, a distinct disadvantage that led to their lowest frequencies.

The double-dose disadvantage explained the low frequencies of GLU, LGU, UGL, ULG, LGL, GLG, LGG, GGL, GLL, and LLG, but not the low frequencies of LUG and GUL which fell into class III even though they did not incur any double-dose copy number changes, in contrast to GUG and LUL which belonged to the more abundant class II. The contrast indicates that lineage effects were important not only to LOH partitions (Figs. 1b, 3b, and 5b) but also to the frequencies of different CNV orders. In GUG, the G status of T-stage cells constituted a reversion to the G status of N-stage cells. In LUL, the L status of T-stage cells likewise constituted a reversion to the L status of the N-stage cells. Thus, both these reversions were favored by lineage effects, allowing GUG and LUL to join class II even though they each incurred two CNV status changes. In contrast, lineage effects acted against LUG and GUL, because the CNV status of the T-stage cells in these cases was not a restoration of the CNV status of the N-stage cells, thereby explaining their diminished frequencies. These lineage effects were observable when different sizes of sequence windows were employed for CNV identification: as shown in Fig. 8c, both the quotients (*Q* value) of GUG/GUL and LUL/LUG greatly exceeded unity (marked by dashed red line), yielding significant lineage effects of *p* < 10^−16^ for all sizes of sequence windows ranging from 50 to 500 kb.

## Discussion

Premalignant and precancer cells have been observed in a variety of cancers [1–14]. The clonal evolution hypothesis of tumor cell populations postulates that a common progenitor normal cell gives rise to both precancer cells and tumor cells through stepwise genetic variations in cancer evolution [1]. Within this conceptual framework, the relationship between a precancer stage cell and the tumor cell may vary between an “inverted pyramid” mode where there exists a large degree of interdependence between successive mutations such that the early mutations provide internal selection pressures for later mutations to result in a linear selection of mutations and a “nexus” mode where the mutations are not interdependent and there are no selection pressures, and the emergence of precancer cells and tumor cells would proceed largely in parallel [45]. In the present study, genomic sequence analysis employing the AluScan platform revealed that the nontumor tissue isolated at > 2 cm from the tumor and P-stage tissue isolated at ≤ 2 cm from tumor’s edge (see the “Methods” section) both contained numerous GOHs and LOHs along with CNVs, thus pointing to these tissue regions as premalignant or early malignant stages despite their apparently normal cell morphologies. Three lines of evidence support a largely sequential relationship between the stages of cancer genome development, leading from the germline B-stage genome to the N-stage genome, then the P-stage genome, and finally the T-stage genome, and beyond that in certain instances to an M-stage genome:

- Residue-by-residue tracing of these mutations through the B-, N-, P-, and T-stages pointed to much higher LOH rates and GOH-m rates than GOH-M rates (Fig. 1a), in accord with our earlier findings [17]. In addition, LOH reversals of the forward GOH mutations with strong lineage effects on the partition of LOH products of heterozygous residues between the homozygous MM and mm genotypes were prevalent, favoring the restoration of the homozygous germline genotypes that gave rise to the heterozygous residues in the first place. These lineage effects, observable not only in the aggregate for the B-N-P-T samples (highlighted by yellow triangles in Fig. 1b) but also for individual B-N-P-T samples (Additional file 3: Table S3), were in accord with the linear inverted pyramid relationship, with GOH mutations occurring at the N-stage exerting substantial influence on the outcome of LOH mutations occurring at the P-stage and GOHs occurring at the P-stage exerting substantial influence on the outcome of LOH mutations occurring at the T-stage. In contrast, such effects would be difficult to explain based on the nexus-type relationship arising from parallel developments of the N-, P-, and T-stage cells from the common progenitor normal cell.
- The diminishing LOH-M/LOH-m ratios from L13/L14 = 5.7 to L15/L16 = 2.9 and on to L21/L22 = 1.7, again also observable with both aggregate (Fig. 1b) and individual (Additional file 3: Table S3) B-N-P-T samples, might stem from the longer period of positive selection for revertant mutations the N-stage cells were subjected to compared to P-stage cells and P-stage cells compared to T-stage cells. In any event, the sequential change in the LOH-M/LOH-m ratio from N to P and then to T was readily explicable in terms of the inverted pyramid mode of sequential selection but not in terms of the nexus mode of parallel selection.
- While the steeply unequal CNV frequencies between GUG and GUL and between LUL and LUG (Fig. 8c) were again in accord with the sequential inverted pyramid mode of development from N to P and then to T, they would be incompatible with a parallel nexus mode.

**Fig. 9:**
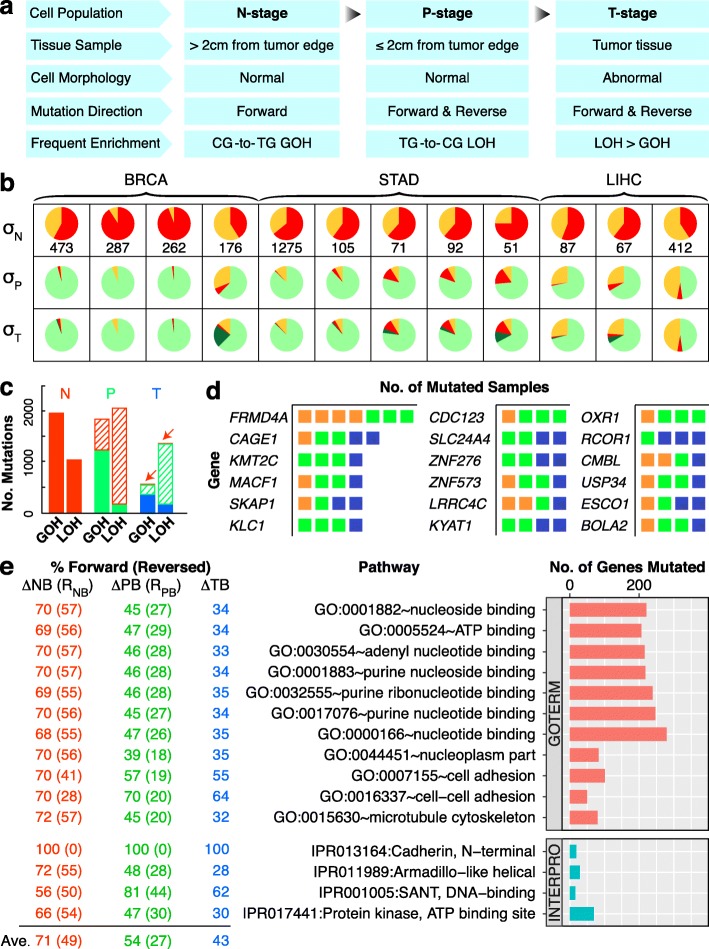
Mutational features of different stages of cancer development. **a** stage-specific population (SSP) model of cancer development. **b** Reversal of N-stage SNVs at P- and T-stages in 12 tetra sample cases of BRCA, STAD, and LIHC. Colored sectors represent N-stage GOHs (red), N-stage LOHs (orange), P-stage reversals of N-stage SNVs (light green), and T-stage reversals of N-stage SNVs (dark green), respectively. *σ*_N_ shows the total number of SNVs occurred at N-stage compared to B-stage cells with relative proportions of GOH and LOH shown by colored sectors. *σ*_P_ and *σ*_T_ show the proportions of N-stage SNVs unreversed and reversed at P-stage and T-stage, as color-coded sectors. **c** Forward and reverse SNVs in N-, P-, and T-stage samples. Left: N-stage SNVs comprising forward GOHs and LOHs (solid red). Middle: P-stage SNVs comprising forward GOHs and LOHs (solid green) and GOHs and LOHs that reversed N-stage LOHs and GOHs, respectively (striped red). Right: T-stage SNVs comprising forward GOHs and LOHs (solid blue) and GOHs and LOHs that reversed P-stage LOHs and GOHs (striped green) and N-stage LOHs and GOHs (striped red highlighted by arrows), respectively. **d** Frequently mutated genes in the 12 tetra sample cases. Each mutation found in a BRCA (orange), STAD (green), or LIHC (blue) sample is represented by a square listed with the gene. **e** Pathway enrichments of mutations in the 12 tetra sample cases. The horizontal bars showing the numbers of mutated genes are arranged from top down in the order of increasing Bonferroni-corrected *p* values. The percentage of genes in the pathway that displayed one or more forward SNVs at N-, P-, or T-stage is indicated under ∆NB, ∆PB, and ∆TB, respectively. The percentage of N-stage SNVs that were reversed subsequently at P- or T-stage was indicated by (R_NB_). The percentage of P-stage SNVs that were reversed subsequently at T-stage was indicated by (R_PB_) (see Fig. 1 for abbreviations)

Accordingly, these convergent lines of evidence derived from the B-N-P-T tetra samples, and also supported by those from the B-N-T trio samples (Fig. 3), the N-T-M trio samples based on AluScan, WES, and WGS (Figs. 5 and 6), and ICGC B-T-paired samples based on WGS (Fig. 7), pointed to a linear cancer development sequence between the B-N-P-T stages, as represented in the stage-specific population (SSP) model in Fig. 9a. In this SSP model, while each of the N-, P-, and T-stage cell populations could comprise multiple cell clones, a majority of the cell clones within the same stage would display largely similar mutational and morphological characteristics.

Figure 9b shows that although the 12 B-N-P-T tetra sample cases were derived from the three types of solid tumors, they all displayed a largely similar mutational trend; the SNVs found at N-stage (*σ*_N_) comprised different proportions of GOHs (red) and LOHs (orange) and underwent substantial reversions in P-stage cells (light green), followed by a much smaller number of additional reversions occurring in the T-stage cells (dark green). Altogether, that more N-stage SNVs were reversed in P-stage than in T-stage in all 12 samples amounted to a highly non-random observation (*p* = 3.1 × 10^−12^), in confirmation of the stage-specific difference in mutational activities between P- and T-stage cells. The LOH/GOH ratios in tumor tissues relative to same-patient white blood cell samples as controls were different for different cancer types: 0.32 for BRCA, 1.16 for STAD, and 4.29 for LIHC (data from Additional file 4: Table S4), in accordance with Fig. 7b which showed more LOHs in LIHC than in NSCLC or ICC. Importantly, as shown by a comparison of Additional file 11: Figure S2 with Figs. 5a–b and 6a, WGS sequences and their AluScan-capturable subsets of the N-T-M trio samples in the WGS-Liver-M group were in substantial accord with respect to the finding of forward-reverse mutation cycles (FR-cycles) between different cancer developmental stages, the greater LOH rates than GOH rates in the N-to-T transition, the strong lineage effects observed in the LOH-reversals in the T-to-M transition, and the mutational profiles of both the N-to-T and T-to-M transitions. These results provide useful validation for the application of the AluScan platform for mutation analysis.

Earlier, comparison of different cancer-control pairs indicated that the LOH rates were greater than GOH-m rates, which were in turn greater than GOH-M rates in different types of cancers, pointing to widespread interhomolog chromosomal gene conversions arising from defective DNA double-strand break repair in cancers to cause massive LOHs and tag-along GOHs [17]. In addition, the prominence of “signature 1A” in Fig. 1d suggests that N-stage cells acquired substantial numbers of GOHs through deamination of 5-methylcytosine. In Fig. 1b, likewise, the LOH rate of 0.04 in the L13 step exceeded the GOH-m rate of 0.02 in the G6 step, and even more so the GOH-M rate of 1.8E−5 in step G1. This high propensity of N-stage cells toward LOH mutations suggest that they already resembled mature cancer cells in the possession of a defective DSB repair that allowed massive gene conversions, thus establishing the gene conversion-enhancing DSB defect as a very early event in cancer development and a key departure of N-stage cells from normal B-stage cells. The major consequence of this defect was massive forward LOHs and tag-along GOHs. On the one hand, these large numbers of LOHs and GOHs would increase the probability of generating essential mutations needed by the developing cancer cells to advance toward full-fledged malignancy. On the other hand, they could also bring about excessive mutation load that would slow down the growth of the increasing propagation-unconstrained cells. Accordingly, to reduce the mutation load, mutations of the N-stage genome that served to reverse the forward LOHs and GOHs would be positively selected resulting in high reversal rates of 0.96 for L1 and L8 steps, 0.56 for G11 step, and 0.75 for G12 step, thereby conferring on the P-stage cells their outstanding characteristics of highly active reversals. Notably, in the B-N-P-T tetra samples, a great majority of both the forward and reverse LOHs occurred within the copy number neutral regions (Additional file 14: Figure S4), suggesting that both the forward LOHs and the reverse LOHs were mostly caused by DSB repair through gene conversion. While the different types of cancers analyzed showed similar N- and P-stage mutational properties, different cancers varied with respect to the abundance of LOH relative to GOH in both the AluScan results (Fig. 9b) and the WGS results (Fig. 7b), exemplified by the higher LOH frequencies in LIHC compared to other types of cancers and suggesting that LOH/GOH ratios could be useful for cancer subtyping. The finding of high rates of SNVs in the N-stage cells despite their normal morphology was consistent with the elevated SNV prevalences by 27-fold (*p* < 0.001) or 36-fold (*p* < 0.0001) observed in normal kidney cortices of the subjects that were smokers or exposed to the environmental carcinogen aristolochic acid, respectively [46].

The necessity of mutation load reduction in cancer development was also consistent with the slower multiplication of cultured transformed cells than untransformed cells at low population density, e.g., upon transformation of a C3H/10T1/2CL8 fibroblast cell line derived from C3H mouse embryos by 3-methylcholanthrene, the transformed cells exhibited a saturation density 2–3 times that of untransformed cells, but generation times of 22 and 27 h, viz. 40–70% longer than the 15.5 h for the untransformed cells [47]. Likewise, NIH 3T3 cells displayed retarded growth at low density and increased saturation density preceding the formation of transformed loci [48], while the increased density attained might stem from reduced contact inhibition, and the longer generation times could be the result of excessive deleterious mutations.

The pronounced reversions of N-stage SNVs in P-stage, P-stage SNVs in T-stage, and T-stage SNVs in M-stage (Figs. 1b, 3b, and 5b) suggest that the FR mutations, or FR-cycles, between successive development stages could be a common cellular evolution strategy for the adaptation to the changes encountered during stage transitions. This was confirmed by the results in Fig. 4, where adaption of HeLa cells to replating and growth likewise gave rise to FR-cycles comprising a major wave of CG>TG rich GOHs around day 10 followed by reversions via TG>CG rich LOHs later around day 14. The similarity between the FR-cycles of the developing cancer cells in the patch diagrams of Fig. 1 and the FR-cycles of the serially sampled HeLa cells in the patch diagrams of Fig. 4b was evident in the strong lineage effects of the reverting LOH mutations as highlighted by yellow triangles in both instances, as well as the close agreement between the rates of both the GOH steps (G1 and G6 in Fig. 1b, the equivalent G1 and G3 in Fig. 4b) and the ensuing LOH steps (L1, L2, L7, and L8 in Fig. 1b, and the equivalent L1, L2, L3, and L4 in Fig. 4b) observed in the two sets of patch diagrams. Since the HeLa samples were taken from the time series of cell populations on days 5, 10, and 14, the similarity of the FR-cycles between the N-P-T stages and those between different HeLa cell samplings suggest that, in both cases, a wave of GOH mutations was followed subsequently by extensive reversions. Moreover, it is notable that, when a deviant *Drosophila melanogaster* population induced by extreme starvation was allowed to readapt to the ancestral culture environment, reversions of SNPs back to ancestral allele genotypes over 50 generations of evolution amounted to about 50% [49], comparable to the average ~ 39% level of SNV reversions exhibited by P-stage cells in the form of type IVa changes in Fig. 2c.

The *FRMD4A* gene [50] was mutated in 4 BRCA cases and 3 STAD cases in the 12 tetra sample cases, and *CAGE1* for cancer antigen 1 [51] was mutated in BRCA, STAD, and LIHC samples. SNVs recurrent in 4 out of 12 cases were detected for 16 different genes (Fig. 9d). Based on the GOTERM and INTERPRO databases, pathway enrichment analysis shows that SNVs in the B-N-P-T tetra samples were frequent in the cell adhesion pathway (Fig. 9e). It was striking that all of the N-stage mutations in the cadherin N-terminal domain family persisted unreversely throughout the P- and T-stages, pointing to the importance of this family of cell adhesion molecules at multiple stages of cancer development, see Additional files 16 and 17: Tables S12 and S13 for mutated genes and pathways.

## Conclusion

In conclusion, the occurrences of a wave of forward mutations followed by their reversals are observed in both cancer development samples and serial samples of cultured HeLa cells. Because cancers are driven by mutations, the nature of the mutations in the evolving cancer cells furnishes an appropriate basis for delineating the major stages of carcinogenesis. In the present study, the mutational profiles of the cell populations in the N-, P-, and T-stage samples showed that N-stage cells surprisingly harbored large numbers of SNV mutations, more GOHs than LOHs, which were enriched with NCG>NTG type of GOHs with associated CNVs. The P-stage cells displayed, relative to N-stage cells, more LOHs than GOHs. A major fraction of their LOHs represented reversals of the forward GOH mutations found in N-stage and was enriched with NTG>NCG type of LOHs with associated CNVs. In the T-stage cells, the ratio between LOHs and GOHs was even higher than P-stage cells (Fig. 9c, see Additional file 3: Table S3 for data used in this plot). At T-stage, there were numerous reversals of P-stage mutations but far fewer reversals of N-stage mutations. The extents of these reversals of N-stage mutations in P-stage and P-stage mutations in T-stage were unexpectedly large. Moreover, as shown by the AluScan, WGS, and WES results in Fig. 5b, the uniformly high rates of the revertant L1, L4, G5, and G6 steps in the different groups of cancers indicated that T-stage mutations were likewise subjected to extensive reversals in M-stage cells, which confirmed the importance of FR mutations as a cellular mechanism for regulating the mutation load. Accordingly, the N-, P-, and T-stage cell populations represented different developmental stages of cancer development, each with its own mutational characteristics that best fulfilled the role of that particular developmental stage. The identification of the intermediate N- and P-stages not only provides a basis for facilitating early diagnosis, subtyping, and staging of cancers, but also suggests that the early N-stage cells, which have not yet accomplished their requisite mutation reversals and hence mutation load reduction, might be relatively deficient in growth and replication vigor, in which case it could be advantageous to target therapeutic interventions at these early stages of precancer and cancer cells before they have accomplished their mutation reversals to become fully malignant, therapy-resistant cancers.

## Additional files

Additional file 1: **Table S1.** Information on 103 samples analyzed by AluScan. (XLSX 13 kb)

Additional file 2: **Table S2.** Tumor purities of B-N-P-T tetra and B-N-T trio samples estimated by absCN-seq. (XLSX 10 kb)

Additional file 3: **Table S3.** Summary of SNV mutations in B-N-P-T tetra samples. (XLSX 14 kb)

Additional file 4: **Table S4.** The exact residue-by-residue SNV mutations in each sample of the B-N-P-T tetra sample cases. (XLSX 652 kb)

Additional file 5: **Figure S1.** Total numbers of different dinucleotide sites in the human genome. Numbers of CG as well as other 15 types of dinucleotides in human reference genome hg19 are plotted out. (PDF 100 kb)

Additional file 6: **Table S5.** Summary of CNV mutations in B-N-P-T tetra samples. (XLSX 11 kb)

Additional file 7: **Table S6.** Summary of SNV mutations in B-N-T trio samples. (XLSX 14 kb)

Additional file 8: **Table S7.** The exact residue-by-residue SNV mutations in each sample of the B-N-T trio sample cases. (XLSX 3018 kb)

Additional file 9: **Table S8.** Summary of SNV mutations in N-T-M trio samples. (A) AluScan, (B) WES-Non-Lung, (C) WES-NSCLC-L, (D) WES-NSCLC-H, and (E) WGS-Liver-M. (XLSX 48 kb)

Additional file 10: **Table S9.** The exact residue-by-residue SNV mutations in each sample of the AluScan N-T-M trio sample cases. (XLSX 214 kb)

Additional file 11: **Figure S2.** SNV mutations in the AluScan-capturable regions of WGS samples in the WGS-Liver-M group. In this figure, AluScan-capturable sequences, corresponding to all the AluScan-captured sequences analyzed in Fig. 1, were extracted from the four N-T-M trio sets in WGS-Liver-M group and analyzed. **a** Genotypic changes in T-stage and M-stage cells. The numbers of genotypic changes in T- or M-stage sequences relative to N-stage sequences are represented by ΔTN and ΔMN, respectively. **b** Patch diagrams tracing SNVs between the N-, T-, and M-samples. **c** Mutational profiles for the ∆TN and ∆MT SNV changes as numerically indicated in the patch diagrams in part b. In each vertical bar in the ∆TN tier, the solid segment represents the SNVs that were reversed in the ∆MT tier, whereas the open segment indicates the unreversed SNVs. (PDF 1230 kb)

Additional file 12: **Table S10.** Numbers of GOHs and LOHs in 22 ICC, 86 LIHC, and 75 NSCLC samples. (XLSX 14 kb)

Additional file 13: **Figure S3.** Mutation-rate diagrams of Pilot-63 samples from ICGC analyzed by WGS. **a** Mutation-rate diagrams for GOHs. Each of the ten diagrams of triplet duplexes corresponds to a context group, labeled 1–10 as in Fig. 7d. The mutation rates of opposing GOH mutations are labeled on double-headed arrows, except for the single-headed curved arrows in groups 7–10, where the two sequences are identical in a triple duplex. Each double-headed arrows is accompanied by two color-coded mutation rates that correspond to the heights of color-coded bars in Fig. 7d, e.g., in context group 1, the conversion of double-stranded ACA/TGT to AAA/TTT is associated with a mutation rate of 162, colored red to correspond to the red C>A bar with A.A context in the left panel of Fig. 7d; whereas the opposing conversion of AAA/TTT to ACA/TGT is associated with a mutation rate of 641, colored orange to correspond to the orange A>C bar with A.A context in the left panel of Fig. 7d. **b** Mutation-rate diagrams for LOHs. The arrows employed are similar to those in part a. All arrows in parts a and b are shown as dashed lines for transitions (TSs) or solid lines for transversions (TVs). In the ten diagrams in part a or part b, the boxed TS/TV ratio given for each diagram represents the ratio pertaining to all the TS and TV mutations in the diagram, e.g., in diagram 1 of part a, TS equals the sum of the four TS rates in the diagram, and TV the sum of the eight TV rates, yielding TS/TV = 1430/2804 = 0.51. The different rates in the diagrams in parts a and b are color-coded as in Fig. 7d. (PDF 459 kb)

Additional file 14: **Figure S4.** Most of the LOHs observed in the course of cancer development occurred in copy-neutral regions of the genome. **a** Upper panel: reverse LOHs occurring in the N-to-P transition (viz. ∆PN). Pie chart indicates that 1850 out of 1875 (98.7%) of the reverse LOHs via L1 and L8 steps analyzed in Fig. 1b occurred in copy-neutral regions. **b** Upper panel: reverse LOHs occurring in the P-to-T changes (viz. ∆TP). Pie chart indicates that 1095 out of 1150 (95.2%) of the reverse LOHs via L3 and L10 steps occurred in copy-neutral regions. **c** Upper panel: forward LOHs occurring in the B-to-N transition (viz. ∆NB). Pie chart indicates that 986 out of 1028 (95.9%) of the forward LOHs via L13 and L14 steps occurred in copy-neutral regions. In parts **a**–**c**, the lower panels show for reference the proportions of CN-neutral, CN-gain, and CN-loss in the course of the B-to-N, N-to-P, and P-to-T transitions, respectively. (PDF 853 kb)

Additional file 15: **Table S11.** The exact window-by-window CNV mutations in each sample of the B-N-P-T tetra sample cases. (XLSX 783 kb)

Additional file 16: **Table S12.** List of genes harboring SNV mutations in B-N-P-T tetra sample cases. (XLSX 317 kb)

Additional file 17: **Table S13.** SNV mutations enriched pathways and genes in B-N-P-T tetra sample cases. (XLSX 109 kb)

## Abbreviations

AluScan: Genome-wide scanning using Alu-based primers
B: White blood cell
BRCA: Breast invasive carcinoma
CNV: Copy number variation
CNVT: Somatic copy number variation
CpGe: Evolutionary conserved CpG doublets
GOH: Gain of heterozygosity
LIHC: Liver hepatocellular carcinoma
LOH: Loss of heterozygosity
M: Metastatic tumor
MeMRE: Unmethylated CpG
N: Nontumor
NSCLC: Non-small cell lung cancer
P: Paratumor
rCNV: Recurrent copy number variation
SNV: Single-nucleotide variation
STAD: Stomach adenocarcinoma
T: Primary tumor
TCGA: The Cancer Genome Atlas
WES: Whole-exome sequencing
WGS: Whole-genome sequencing
