# Supplementary figures and images for: Forward and reverse mutations in stages of cancer development
Source: Hum Genomics. 2018 Aug 22;12:40. doi: 10.1186/s40246-018-0170-6 (PMC6104001; doi:10.1186/s40246-018-0170-6)

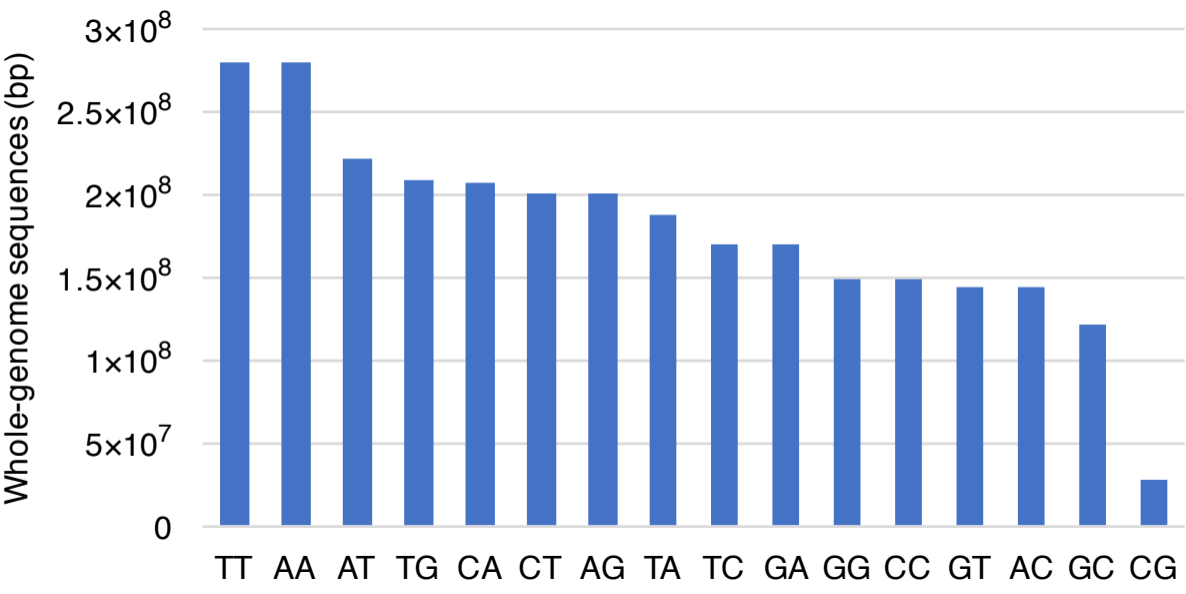

Supplement: Supplementary file 5 — Figure S1. Total numbers of different dinucleotide sites in the human genome. Numbers of CG as well as other 15 types of dinucleotides in human reference genome hg19 are plotted out. (PDF 100 kb) [file 40246_2018_170_MOESM5_ESM.pdf]

|    | N      | $\Delta$ TN | $\Delta$ MN |       |
|----|--------|-------------|-------------|-------|
| MM | 1.1E+8 | 229         | 190         | GOH-M |
| mm | 32874  | 8           | 5           | GOH-m |
| Mm | 34185  | 473         | 210         | LOH   |

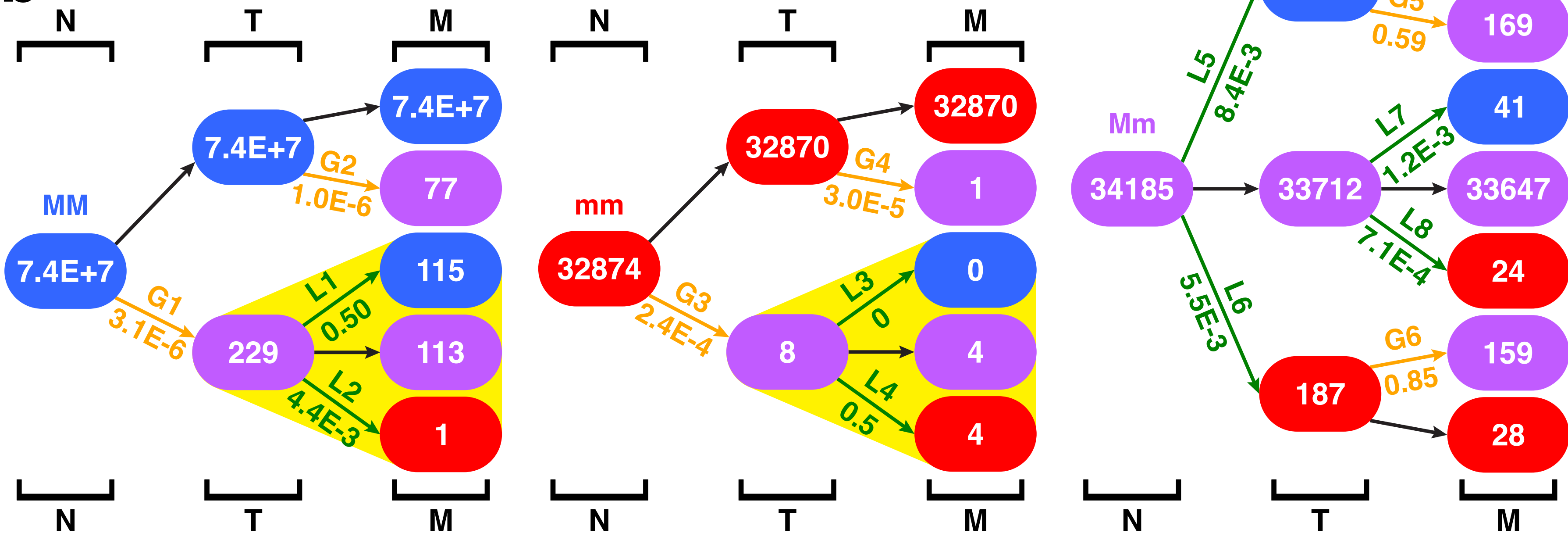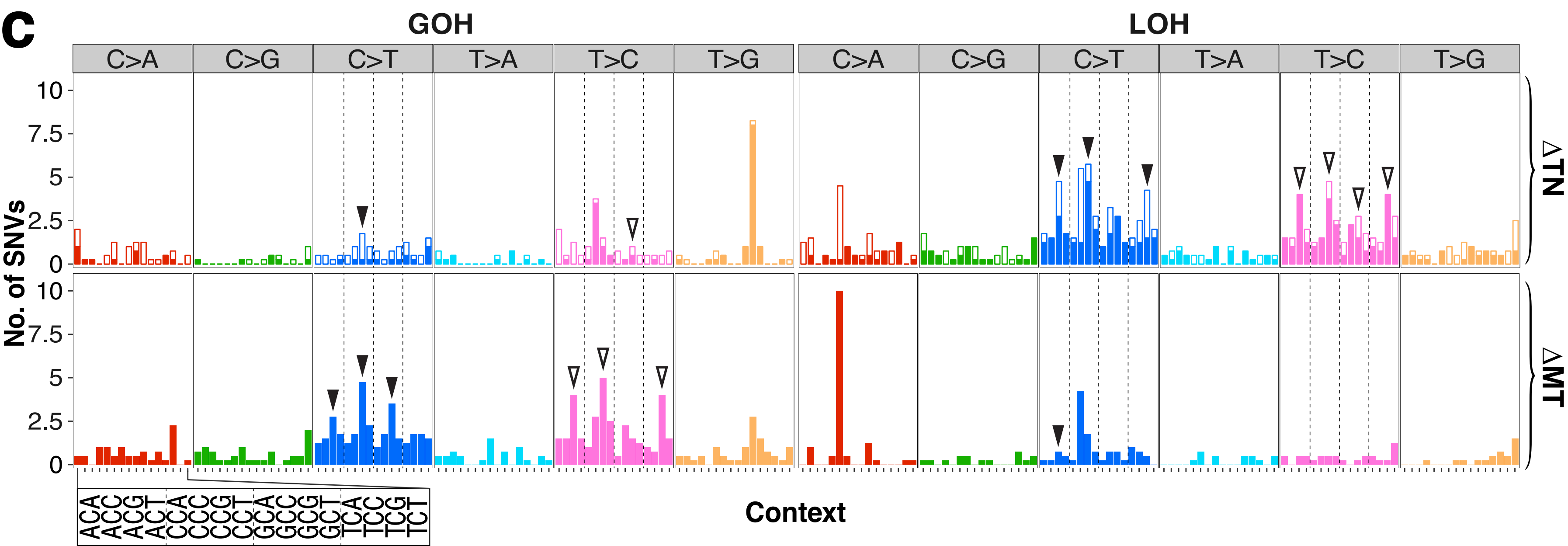

Supplement: Supplementary file 11 — Figure S2. SNV mutations in the AluScan-capturable regions of WGS samples in the WGS-Liver-M group. In this figure, AluScan-capturable sequences, corresponding to all the AluScan-captured sequences analyzed in Fig. 1, were extracted from the four N-T-M trio sets in WGS-Liver-M group and analyzed. a Genotypic changes in T-stage and M-stage cells. The numbers of genotypic changes in T- or M-stage sequences relative to N-stage sequences are represented by ΔTN and ΔMN, respectively. b Patch diagrams tracing SNVs between the N-, T-, and M-samples. c Mutational profiles for the ∆TN and ∆MT SNV changes as numerically indicated in the patch diagrams in part b. In each vertical bar in the ∆TN tier, the solid segment represents the SNVs that were reversed in the ∆MT tier, whereas the open segment indicates the unreversed SNVs. (PDF 1230 kb) [file 40246_2018_170_MOESM11_ESM.pdf]

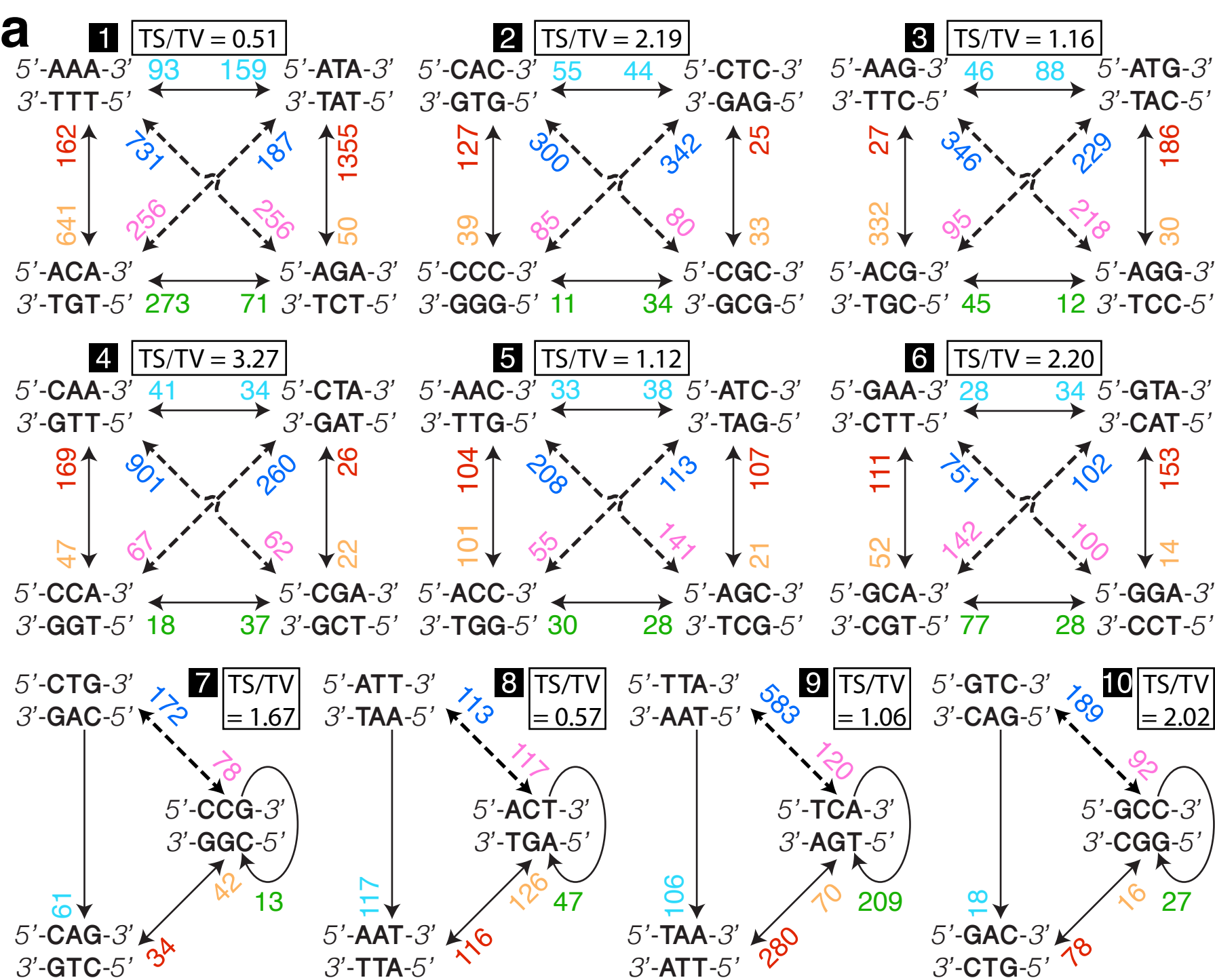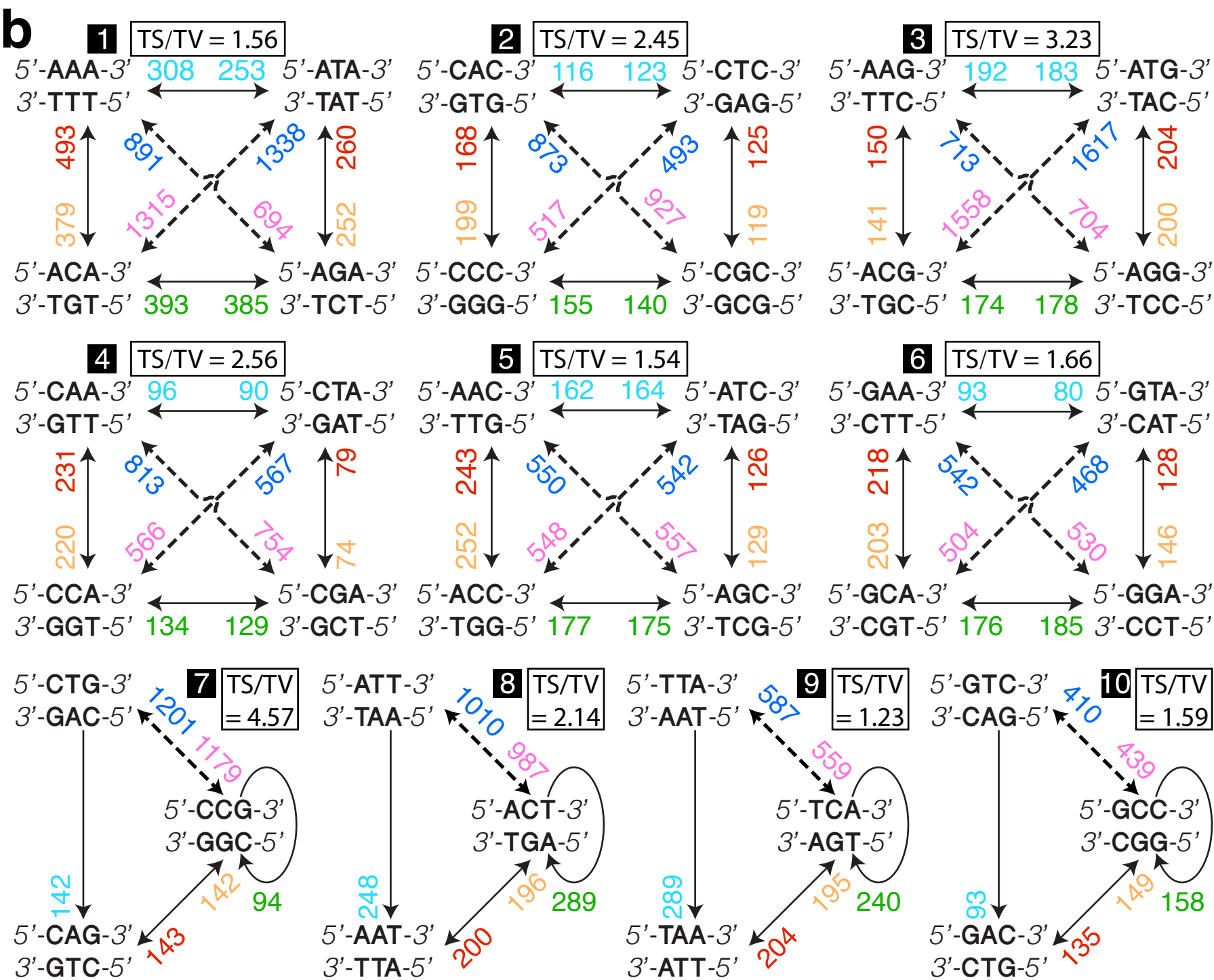

Supplement: Supplementary file 13 — Figure S3. Mutation-rate diagrams of Pilot-63 samples from ICGC analyzed by WGS. a Mutation-rate diagrams for GOHs. Each of the ten diagrams of triplet duplexes corresponds to a context group, labeled 1–10 as in Fig. 7d. The mutation rates of opposing GOH mutations are labeled on double-headed arrows, except for the single-headed curved arrows in groups 7–10, where the two sequences are identical in a triple duplex. Each double-headed arrows is accompanied by two color-coded mutation rates that correspond to the heights of color-coded bars in Fig. 7d, e.g., in context group 1, the conversion of double-stranded ACA/TGT to AAA/TTT is associated with a mutation rate of 162, colored red to correspond to the red C>A bar with A.A context in the left panel of Fig. 7d; whereas the opposing conversion of AAA/TTT to ACA/TGT is associated with a mutation rate of 641, colored orange to correspond to the orange A>C bar with A.A context in the left panel of Fig. 7d. b Mutation-rate diagrams for LOHs. The arrows employed are similar to those in part a. All arrows in parts a and b are shown as dashed lines for transitions (TSs) or solid lines for transversions (TVs). In the ten diagrams in part a or part b, the boxed TS/TV ratio given for each diagram represents the ratio pertaining to all the TS and TV mutations in the diagram, e.g., in diagram 1 of part a, TS equals the sum of the four TS rates in the diagram, and TV the sum of the eight TV rates, yielding TS/TV = 1430/2804 = 0.51. The different rates in the diagrams in parts a and b are color-coded as in Fig. 7d. (PDF 459 kb) [file 40246_2018_170_MOESM13_ESM.pdf]
