# Supplementary material for: Forward and reverse mutations in stages of cancer development
Source: Hum Genomics. 2018 Aug 22;12:40. doi: 10.1186/s40246-018-0170-6 (PMC6104001; doi:10.1186/s40246-018-0170-6)
Supplement: Supplementary file 14 — Figure S4. Most of the LOHs observed in the course of cancer development occurred in copy-neutral regions of the genome. a Upper panel: reverse LOHs occurring in the N-to-P transition (viz. ∆PN). Pie chart indicates that 1850 out of 1875 (98.7%) of the reverse LOHs via L1 and L8 steps analyzed in Fig. 1b occurred in copy-neutral regions. b Upper panel: reverse LOHs occurring in the P-to-T changes (viz. ∆TP). Pie chart indicates that 1095 out of 1150 (95.2%) of the reverse LOHs via L3 and L10 steps occurred in copy-neutral regions. c Upper panel: forward LOHs occurring in the B-to-N transition (viz. ∆NB). Pie chart indicates that 986 out of 1028 (95.9%) of the forward LOHs via L13 and L14 steps occurred in copy-neutral regions. In parts a–c, the lower panels show for reference the proportions of CN-neutral, CN-gain, and CN-loss in the course of the B-to-N, N-to-P, and P-to-T transitions, respectively. (PDF 853 kb) [file 40246_2018_170_MOESM14_ESM.pdf]

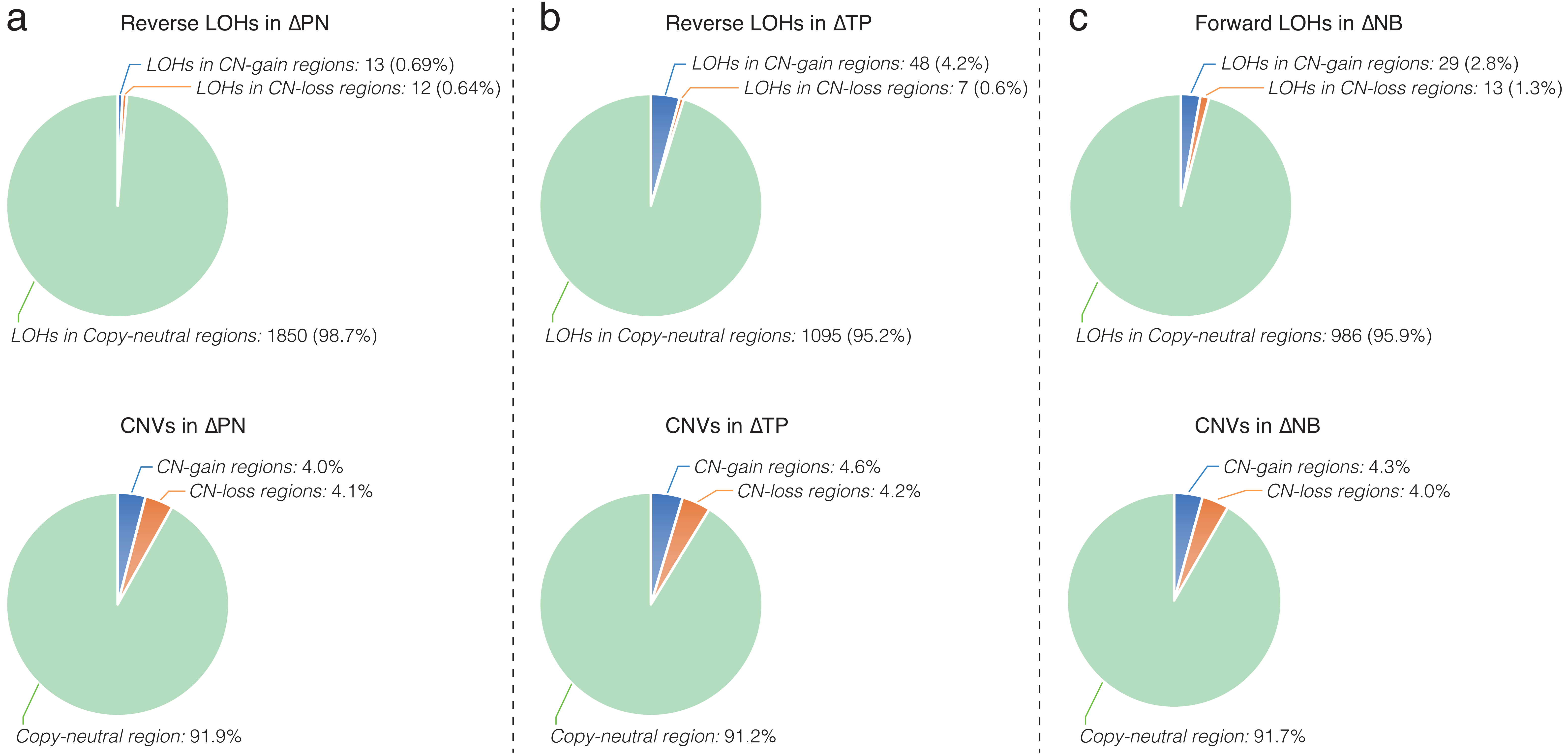
a
Reverse LOHs in ΔPN
LOHs in CN-gain regions: 13 (0.69%)
LOHs in CN-loss regions: 12 (0.64%)
LOHs in Copy-neutral regions: 1850 (98.7%)
CNVs in ΔPN
CN-gain regions: 4.0%
CN-loss regions: 4.1%
Copy-neutral region: 91.9%
b
Reverse LOHs in ΔTP
LOHs in CN-gain regions: 48 (4.2%)
LOHs in CN-loss regions: 7 (0.6%)
LOHs in Copy-neutral regions: 1095 (95.2%)
CNVs in ΔTP
CN-gain regions: 4.6%
CN-loss regions: 4.2%
Copy-neutral region: 91.2%
c
Forward LOHs in ΔNB
LOHs in CN-gain regions: 29 (2.8%)
LOHs in CN-loss regions: 13 (1.3%)
LOHs in Copy-neutral regions: 986 (95.9%)
CNVs in ΔNB
CN-gain regions: 4.3%
CN-loss regions: 4.0%
Copy-neutral region: 91.7%
